# The Conductance and Thermopower Behavior of Pendent *Trans*-Coordinated Palladium(II) Complexes in Single-Molecule Junctions

**DOI:** 10.1021/acsomega.4c06475

**Published:** 2024-08-28

**Authors:** Pablo Bastante, Ross J. Davidson, Wafa Al Malki, Rebecca J. Salthouse, Pilar Cea, Santiago Martin, Andrei S. Batsanov, Colin J. Lambert, Martin R. Bryce, Nicolas Agrait

**Affiliations:** † Departamento de Física de la Materia Condensada C−III, and Instituto Universitario de Ciencia de Materiales “Nicolás Cabrera”, Universidad Autónoma de Madrid, E-28049 Madrid, Spain; ‡ Department of Chemistry, Durham University, Stockton Road, Durham DH1 3LE, U.K.; § Department of Physics, University of Lancaster, Lancaster LA1 4YB, U.K.; ∥ Instituto de Nanociencia y Materiales de Aragón (INMA), CSIC-Universidad de Zaragoza, 50009 Zaragoza, Spain; ⊥ Departamento de Química Física, Universidad de Zaragoza, 50009 Zaragoza, Spain; # Laboratorio de Microscopias Avanzadas (LMA), Universidad de Zaragoza, 50018, Zaragoza, Spain

## Abstract

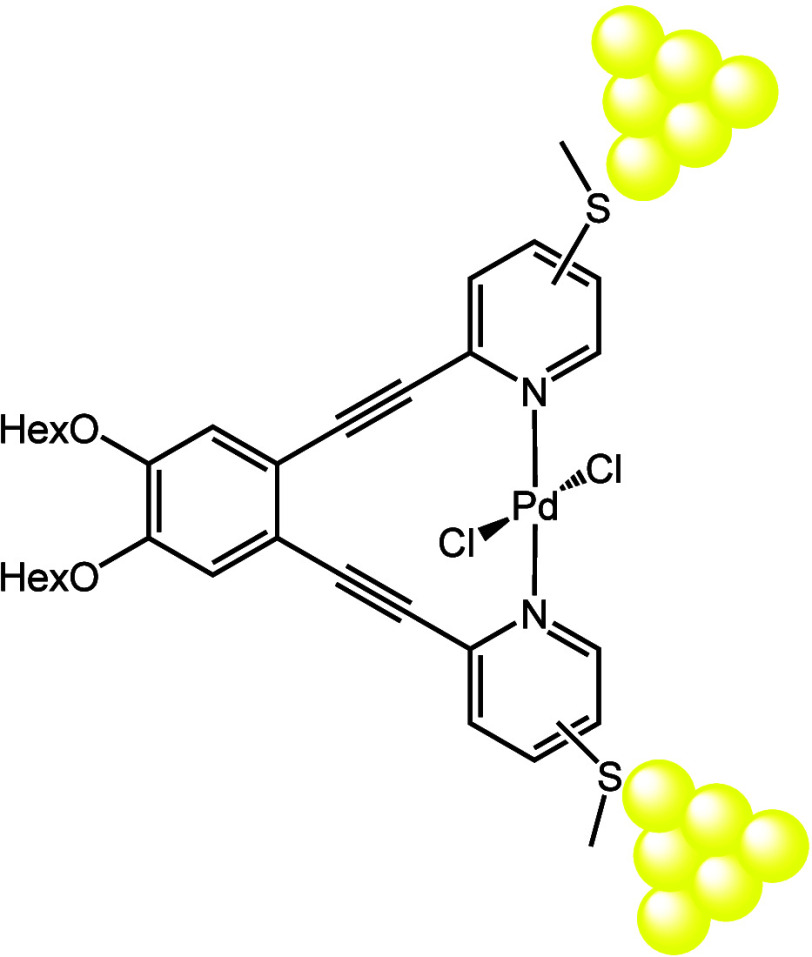

The present work provides insight into the effect of connectivity within isomeric 1,2-bis(2-pyridylethynyl)benzene (bpb) palladium complexes on their electron transmission properties within gold|single-molecule|gold junctions. The ligands 2,2′-((4,5-bis(hexyloxy)-1,2-phenylene)bis(ethyne-2,1-diyl))bis(4-(methylthio)pyridine) (**L^m^**) and 6,6′-((4,5-bis(hexyloxy)-1,2-phenylene)bis(ethyne-2,1-diyl))bis(3-(methylthio)pyridine) (**L^p^**) were synthesized and coordinated with PdCl_2_ to give the *trans*-Pd(**L^m or p^**)Cl_2_ complexes. X-ray photoelectron spectroscopy (XPS) measurements shed light on the contacting modes of the molecules in the junctions. A combination of scanning tunneling microscopy–break junction (STM–BJ) measurements and density functional theory (DFT) calculations demonstrate that the typical lower conductance of *meta-* compared with *para*-connected isomers in a molecular junction was suppressed upon metal coordination. Simultaneously there was a modest increase in both conductance and Seebeck coefficient due to the contraction of the HOMO–LUMO gap upon metal coordination. It is shown that the low Seebeck coefficient is primarily a consequence of how the resonances shift relative to the Fermi energy.

## Introduction

Over the past 20 years, the ability to measure the conductance of individual molecules has facilitated structure–property relationships and a greater understanding of charge transport through a molecule, revealing new quantum phenomena including the major impact of anchor groups and molecular backbone structures on quantum interference.^1−6^ To date, such investigations have been dominated by pure organic conjugated molecules [e.g., oligo(phenylene ethynylenes) (OPEs)] as molecular wires.^7^ However, enhancing the functionality within OPE systems by inclusion of metal ions is alluring owing to the potential to exploit metal–ligand interactions to enhance or gate conductance through external stimuli.^8,9^

The metal complexes that have been measured in a molecular junction can be separated into two groups: (i) those where the metal ion is directly in the linear conductance pathway (e.g., metal acetylides);^10,11^ or (ii) the metal ion is coordinated pendent to the conductive moiety (e.g., 1,10-phenanthroline).^12,13^ The first group demonstrates that the inclusion of the metal can provide conductance enhancement or redox gating,^14−16^ while in the second group the inclusion of the metal impacts the conductance behavior by causing a contraction in the gap between the highest occupied and lowest unoccupied orbitals, i.e. the HOMO–LUMO (H-L) energy gap, in addition to potentially forming Fano resonances.^13^ Despite multiple studies examining the impact of metal coordination on conductance, there is only one report in the literature that directly investigated the impact of the inclusion of metal in a molecular wire on the thermoelectric behavior. This study by Naher et al. using metal acetylide complexes demonstrated that the inclusion of a metal ion [Ru(II) or Pt(II)] within the conjugated backbone results in higher single-molecule conductance *G* and higher Seebeck coefficient *S* compared to pure organic molecular wires. These properties lead to an improvement in the power factor *P*, which represents the ability of a material to extract energy from a thermal difference, according to *P*= *GS*^2^, offering potentially useful molecular electronic properties such as thermoelectric generation or Peltier cooling.^11^ Given that complexes with pendent coordinated metal ions have been shown to have higher conductance, as noted above, we have now investigated whether a similar effect is observed for their thermoelectric behavior.

To test this theory, we employed a 1,2-bis(2-pyidylethynyl)benzene (bpb)-based ligand. This *ortho*-oligo(arylene ethynylene)-type system is highly π-conjugated, facilitating conductance, and undergoes minimal structural changes upon intramolecular coordination of a metal ion to the two terminal pyridyl units. Palladium(II) was chosen as the metal ion as it is a closed-shell species that forms a *trans*-complex with this ligand type with the metal ion in plane with the ligand.^17−19^

## Results and Discussion

**Figure 1:**
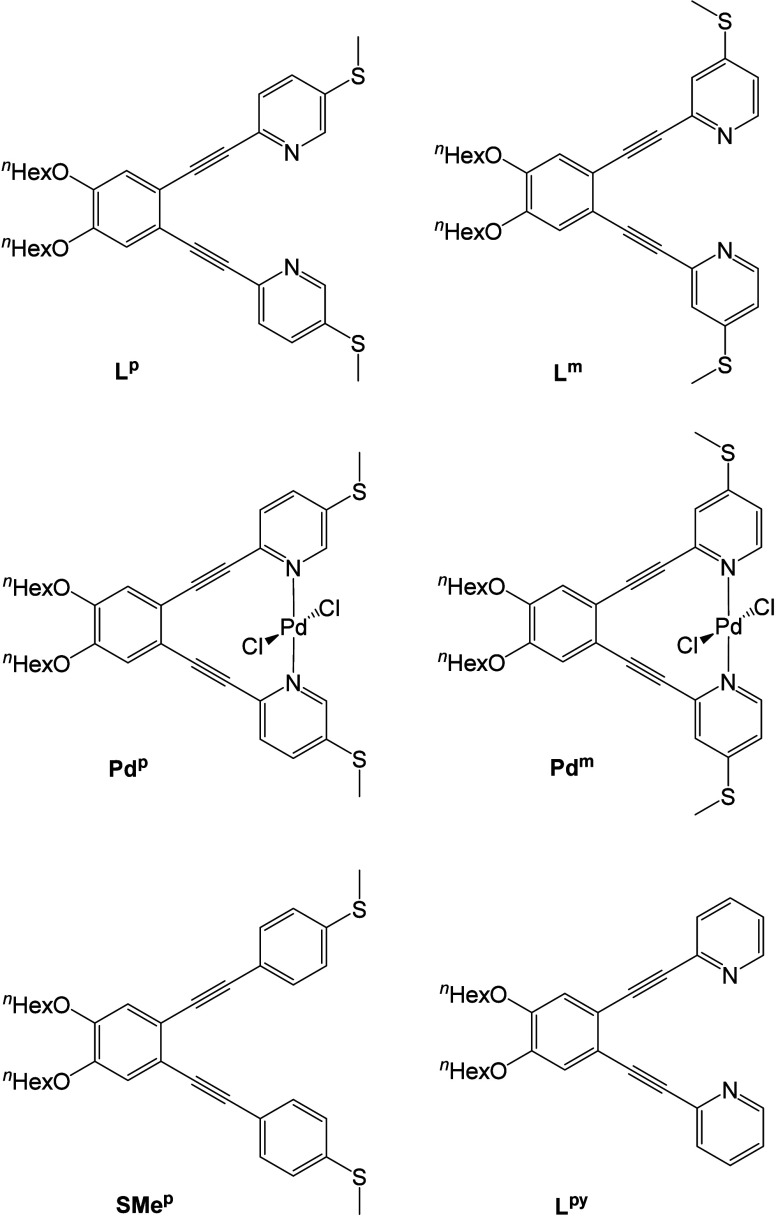
Molecular structures of compounds **L**^**p**^, **L**^**m**^, **Pd**^**p**^, **Pd**^**m**^_,_**SMe**^**p**^, and **L**^**py**^ studied in this work. ^*n*^Hex is *n*-hexyl; *para* and *meta* refer to the positions of the SMe anchors relative to the alkyne bonds.

In order to produce bpb-based complexes that are capable of forming gold|molecule|gold junctions, thiomethyl groups were added to the pyridyl groups, owing to their chemical stability in the presence of metal ions. This was achieved using the approach reported by Li et al.^20^ involving lithiation of the corresponding dibromopyridine in diethyl ether followed by the addition of dimethyl disulfide to give the 2-bromo-thiomethyl-pyridine, which was, in turn, coupled with trimethylsilyl acetylene via Sonogashira protocols followed by *in situ* deprotection and a second Sonogashira coupling to 1,2-bis(hexyloxy)-4,5-diiodobenzene. Ligands with the thiomethyl groups in both *para* (6,6′-((4,5-bis(hexyloxy)-1,2-phenylene)bis(ethyne-2,1-diyl))bis(3-(methylthio)pyridine), **L**^**p**^) and *meta* (2,2′-((4,5-bis(hexyloxy)-1,2-phenylene)bis(ethyne-2,1-diyl))bis(4-(methylthio)pyridine), **L**^**m**^) positions of the pyridine rings provide comparable systems in which the metal ion can be directly involved (*meta*-isomers) or pendent (*para*-isomers) to the conductive path (see Figure 1). Additionally, two reference compounds, namely **SMe**^**p**^ and **L**^**py**^ were also prepared employing analogous Sonogashira coupling reactions. The *n-*hexyloxy groups were attached to the central phenylene ring of all the molecules to ensure good solubility, without sterically hindering the metal complexation or junction formation.

The corresponding *trans*-**PdCl**_**2**_**L**^**p**^ or *trans*-**PdCl**_**2**_**L**^**m**^ was prepared by Hu’s method^21^ of mixing a dichloromethane (DCM) solution of **L**^**p**^ or **L**^**m**^ with an acetonitrile solution of *trans*-PdCl_2_(CH_3_CN)_2_. **Pd**^**p**^ produced crystals suitable by X-ray diffraction using this approach which shows the palladium ion coordinated to both pyridyl groups in a *trans*-square planar fashion with the palladium ion forming a planar complex with the ligand (Figure S19). Unfortunately, **Pd**^**m**^ produced only an amorphous powder.

### Molecular Conductance and Seebeck Coefficient

Conductance measurements were recorded using a home-built scanning tunneling microscope (STM) at ambient conditions.^22^ Samples were prepared from an ∼1 mM solution in dichloromethane (DCM) by depositing the molecules on a preannealed with a propane gas burner Au(99.99% purity)-Cr-glass substrate (Arrandee) for measuring in air after the evaporation of DCM, and from an ∼1 mM solution in mesitylene (98% purity) for measuring in liquid by drop-casting the solution on the gold surface and indenting the tip inside the drop. The tip consisted of a mechanically cut 0.25 mm diameter gold wire (Goodfellow). The molecular junctions were created by performing the STM–BJ technique, measuring the conductance (*G*) and the displacement (*Z*) between the electrodes (tip and sample). Thousands of traces were recorded at a constant bias voltage of 100 mV and the current signal was amplified by a linear current-to-voltage converter with two stages of 10^8^ and 5 × 10^9^ V/A. The series resistance of 2.12 MΩ provided a conductance range from 1.7 *G*_0_ to 1.3 × 10^–6^*G*_0_ with conductance plateaus lower than *G*_0_ observed as the electrical feature of a molecular junction in the recorded traces.

**Figure 2:**
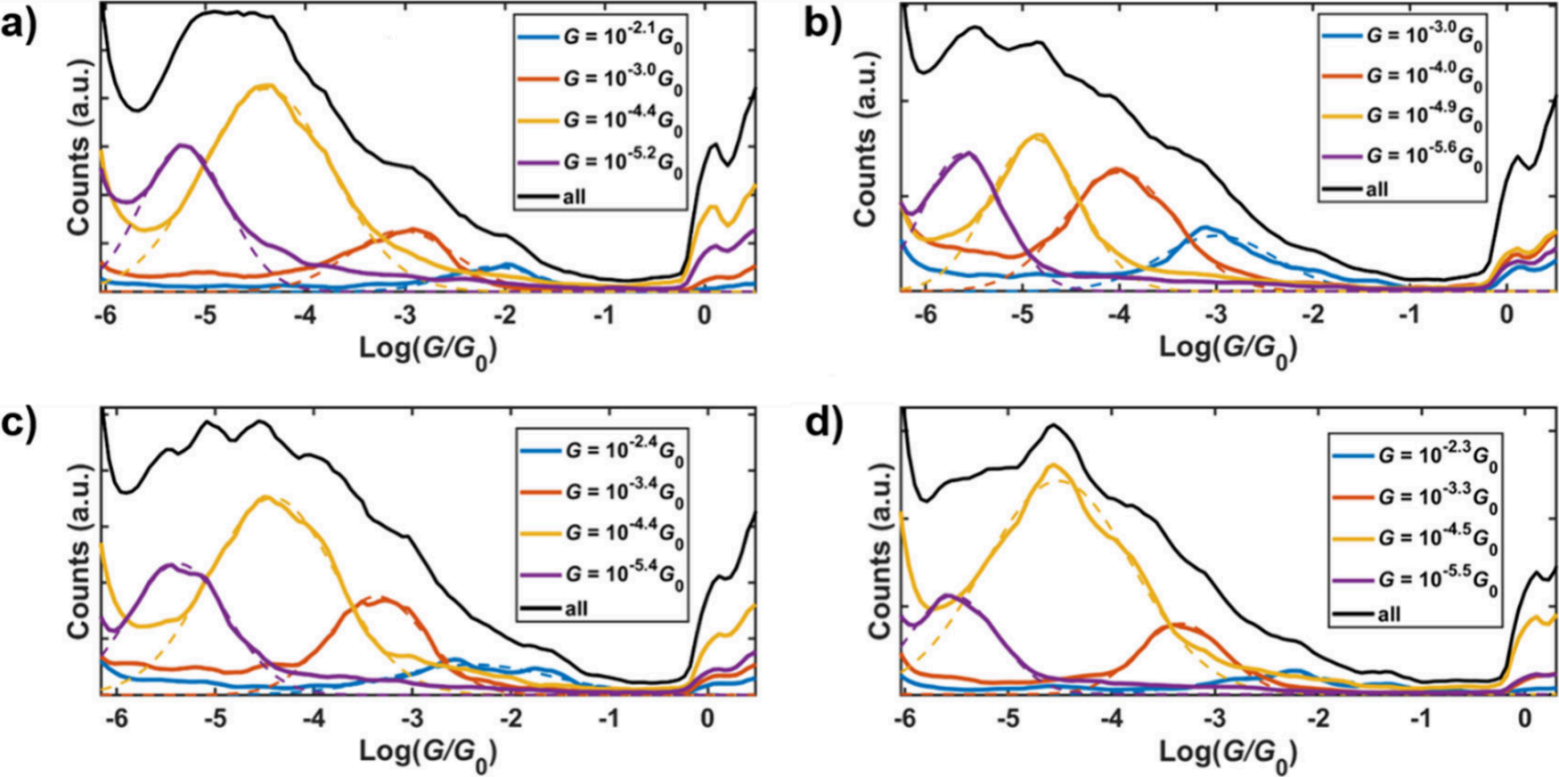
1D conductance histograms of compounds (a) **L**^**p**^, (b) **L**^**m**^, (c) **Pd**^**p**^ and (d) **Pd**^**m**^ respectively, measured in mesitylene solution; separated in conductance classes by colors (the highest conductance peak in blue, then the second highest in red, the main conductance peak in yellow and the lowest conductance peak in purple), each of them fitted to a Gaussian distribution displayed by dashed lines. The black histogram is the total considering all the classes. The legend shows the mean *G* value for each class.

1D histograms were constructed from the conductance measurements correlating the number of measurements recorded at *G* typically giving rise to a distribution around the most probable *G* value. For these compounds, multiple conductance peaks were identified in a large range of *G* values when measured in air suggesting different junction configurations. From all the measured traces, there were excluded “empty” traces showing clean retractions (no features between the contact and the open circuit regimes) and failed retractions due to saturation of the electrical signal, mechanical perturbations, or nonwell-defined breakings. Then, application of an unsupervised machine learning algorithm,^23^ the k-means clustering algorithm,^24^ was used to separate the traces with similar conductance into conductance classes, with a single conductance peak in the histogram. A detailed explanation can be found in the Supporting Information. The resulting conductance classes were fitted independently to a Gaussian distribution determining the mean *G* value of each class given in the legends of Figures 2a-d. Measuring **L**^**p**^, **L**^**m**^, **Pd**^**p**^ and **Pd**^**m**^ under standard methods in air resulted in four distinct conductance classes occurring with approximately equal probability making assignment difficult and further complicated by their similar break-off distances (see Supporting Information Figure S21). Therefore, XPS was employed to examine the molecules on the surface to understand this behavior.

The XPS measurements were carried out on each of these compounds for both a powdered sample and self-assembled monolayers (SAMs) on gold, which were prepared by immersing a gold substrate in a 1 mM solution of the compound in DCM for 48 h. A powdered sample of **L**^**p**^ and **L**^**m**^ in the N 1s region (Figure S43) of the XPS spectrum displayed a peak at 398.5 eV; while for **Pd**^**p**^ and **Pd**^**m**^ the peak appeared at 399.25 eV. In contrast, a SAM of **L**^**p**^ and **L**^**m**^ showed a peak in the N 1s region at 399.2 eV and SAMs of **Pd**^**p**^ and **Pd**^**m**^ also showed the peak at 399.2 eV. All these results suggest that the pyridyl groups for **L**^**p**^ and **L**^**m**^ as well as for **Pd**^**p**^ and **Pd**^**m**^ can contact to the gold substrate providing a new pathway for the conductance. For **Pd**^**p**^ and **Pd**^**m**^ this can be explained through an interchange of the palladium by gold, as demonstrated by the presence of a peak at 340.0 eV attributed to Pd(0), 3p_3/2_, in the Au 4d/Pd 3p region for a SAM of **Pd**^**p**^ and **Pd**^**m**^ (Figure S44).^25^ To corroborate this interaction, a SAM of **L**^**py**^ was prepared under the same conditions and the XPS spectrum in the N 1s region again showed a peak at 399.2 eV, in perfect agreement to that observed for the other SAMs. Therefore, this interaction between the pyridyl groups and the gold substrate could be the origin of the highest conductance features observed in Figure 2, consistent with the behavior proposed by Skipper et al. for the junction assembly of coordination complexes containing transition metal atoms on gold electrodes.^26^

Although it was not possible to identify the specific molecules being formed on the surface, based on literature precedents there are many examples of *ortho*-OPEs reacting with/catalyzed by gold species to annulate,^27−30^ therefore the on-surface formation of a highly conjugated system may account for the high conductance observed. The measurements were repeated in mesitylene solution to reduce the probability of reactions occurring on the surface as solvating the compounds reduces the probability of nonjunction-forming interactions with the gold electrode as well as reducing intermolecular interactions. As a result, the conductance histograms of all the compounds were greatly simplified and although multiple peaks were identified, a dominant conductance feature was observed for each compound (Figure 2 and Table 1). For example, when **L**^**p**^ was measured in air, there was an almost even distribution between conductance peaks, whereas in mesitylene the dominant peak with *G* = −4.4 log(*G*/*G*_0_) accounts for 52.5% of the traces. The reference compounds **SMe**^**p**^ and **L**^**py**^ were measured to confirm that the dominant feature corresponds to an MeS···SMe contacted junction rather than through the pyridyl groups (see Figure S23). **SMe**^**p**^ with terminal phenyl rings instead of pyridyl rings was measured in mesitylene to give a dominant peak at *G* = −4.4 log(*G*/*G*_0_) identical to that of **L**^**p**^, while **L**^**py**^ with no thiomethyl contacts had a dominant peak at a lower *G* = −5.2 log(*G*/*G*_0_), confirming that the dominant conductance class is the MeS···SMe contacted junction when solvated. These conductance classes correspond to the yellow peaks in Figure 2. Additionally, for the lowest conductance class due to their suppression with solvation, lower conductance than the MeS···SMe contacted junction, and comparison to literature examples we assume they are associated with the formation of π-stacked dimers.^31−33^ Although the high conductance classes cannot be assigned, they likely involve the interaction between the pyridyl groups and gold surface. However, the use of solvation lowers their probability of occurring to the extent they can be ignored for the purposes of this study. To our knowledge these are the first conductance measurements to be reported on oligo(arylene ethynylene) derivatives with *ortho* connectivity in the central ring.

**Table 1:** Summary of the Experimental Class III Conductance and Break-off Distance for **L**^**p**^, **L**^**m**^, **Pd**^**p**^, **Pd**^**m**^, and **SMe**^**p**^ Recorded in Mesitylene

	molecule				
	**L**^**p**^	**L**^**m**^	**Pd**^**p**^	**Pd**^**m**^	**SMe**^**p**^
conductance [log(*G*/*G*_0_)]	–4.4	–4.9	–4.4	–4.5	–4.4
break-off distance [nm]	1.31 ± 0.45	1.49 ± 0.21	1.49 ± 0.52	1.41 ± 0.37	1.04 ± 0.39

We can now examine the behavior of these compounds with the assignment of the conductance classes starting with a comparison of the ligands. As discussed above, both **SMe**^**p**^ and **L**^**p**^ have a conductance *G* given by log(*G*/*G*_0_) = −4.4, closer to that of 1,4-bis((4-(methylthio)phenyl)ethynyl)benzene (log(*G*/*G*_0_) = −4.5)^34^ than 2,3-bis((4-(methylthio)phenyl)ethynyl)bicyclo[2.2.1]hepta-2,5-diene ((log(*G*/*G*_0_) = −3.7))^35^ which has an analogous conjugation path, consistent with the proposal of Chen et al. that increasing aromaticity decreases conductance,^36^ although other studies have found no correlation between the extent of aromaticity or antiaromaticity in the backbone and the conductance value.^37−40^**L**^**m**^ has a conductance of *G* = −4.9 log(*G*/*G*_0_) which is lower than **L**^**p**^; this can be attributed to destructive quantum interference (DQI) commonly observed with *meta*-arylene isomers.^4^ However, the difference in conductance of the *para* and *meta* systems became negligible on coordination to palladium, i.e., **Pd**^**m**^ (*G* = −4.5 log(*G*/*G*_0_)) and **Pd**^**p**^ (*G* = −4.4 log(*G*/*G*_0_)). This can be explained by comparing the change in the HOMO–LUMO (H-L) energy gap between the ligands and their respective palladium complexes: Δ|H-L| between **L**^**p**^ and **Pd**^**p**^ is 0.05 eV versus 0.33 eV for **L**^**m**^ and **Pd**^**m**^. Ponce et al. and Chelli et al. demonstrated that the conductance enhancement of coordinating a metal ion to a conductor is dependent on the change Δ|H-L|.^12,13^

In addition to the variation in the conductance behavior, we also observed subtle mechanical differences in the junction for the respective molecules. For the break-off distances, the length of each plateau was considered the higher distance point within a conductance range of *G*_m_ ± 2σ, where *G*_m_ is the mean conductance value of the Gaussian fitting of the conductance histogram and σ is the standard deviation, from the point 0 being the break point of the *G*_0_ contact. With the points from all the traces, a length histogram was built and fitted to a Lorentzian distribution to determine the mean length as the location parameter with an uncertainty described by the scale parameter. Although the break-off distances for the MeS···SMe contacted classes were similar, the distribution was much broader for the *para* molecules, **L**^**p**^ (±0.45 nm) and **Pd**^**p**^ (±0.52 nm) than for the *meta* analogs **L**^**m**^ (±0.21 nm) and **Pd**^**m**^ (±0. 37 nm), because the *meta* series can only adopt a vertical orientation in the junction, while the *para* compounds that “bend” the thiomethyl contact adopt a greater range of angles with the gold electrode.

Switching events can be observed by examining the traces where the conductance abruptly changes among the different conductance values as the STM tip is retracted. These traces represent between the 9% and 16% of the selected traces used to build the conductance histograms depending on the compound, hence they do not affect the statistical analysis. Several possible mechanisms can cause this behavior, namely: the perturbation of a QI feature as the molecule is strained,^41^ slipping of a π-stacked dimer,^42,43^ or changing between tunneling and contacted geometries.^44^ In our case, up to five conductance switching events are observed with changes of up to about 3 orders of magnitude (Figures S39–S42). The metal complexes **Pd**^**p**^ and **Pd**^**m**^ displayed more abrupt events compared to the ligands **L**^**P**^ and **L**^**m**^, possibly because the metal complexes are more rigid and planar than the ligands. The latter is pertinent as the switching usually occurs between the conductance region of the lowest conductance class and the other classes, suggesting that this is associated with the π-stacked dimer. This explanation is consistent with the absence of a switch when the molecules are solvated and the fact that some of the traces are longer than 2 nm, which suggests more than one molecule is involved (the intramolecular S···S distance should be around 1.2–1.4 nm, based on the calculated rotamers). Nevertheless, there are some examples where the lowest conductance region is not involved. In these cases, no more than two switching events are observed, see Figures S39–S42. This is consistent with previously reported results on OPEs and alkanes showing this phenomenon is due to different contact geometries in mechanically controlled break junction experiments.^44^ We have discounted Pd-Py bond breaking as the source of the switching events because they do not occur consistently at a similar distance and are also observed in the free ligands. Additionally, breaking the Pd-Py bond would require straining the ligand, and would imply that the Au-SMe interaction is stronger than the combined Pd-Py bond strength and ligand strain, which is most unlikely.

**Figure 3:**
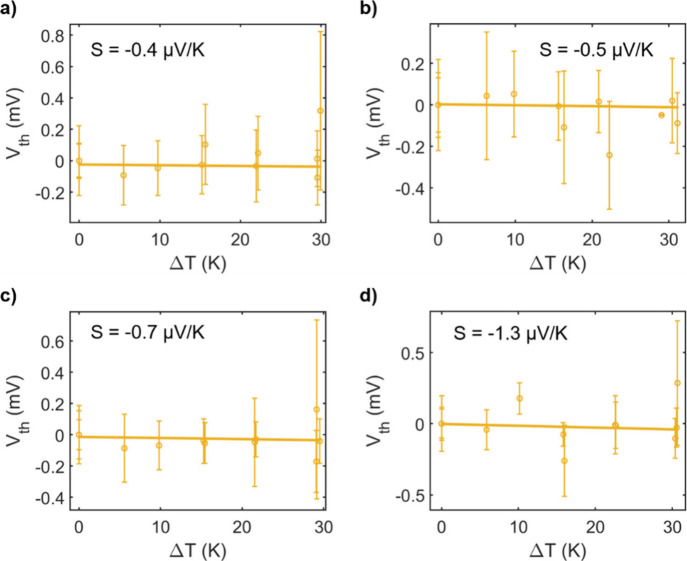
Most probable thermovoltage (V_th_) values with the dispersion as error bars, measured at several temperature differences for the main conductance class of compounds (a) **L**^**p**^, (b) **L**^**m**^, (c) **Pd**^**p**^, and (d) **Pd**^**m**^. The Seebeck coefficient (*S*) is obtained as the slope of the linear regression performed to all data points.

The Seebeck coefficients of the MeS···SMe contacted junctions were measured under ambient conditions to further understand their conductance behavior. For these experiments, the tip was heated with a 1 kΩ resistance implemented on the tip-holder to create a temperature difference between the electrodes (tip and sample) of up to 30 K. Several temperature differences were applied and *IV* curves (±10 mV) were measured simultaneously with the conductance-distance traces.^45^ Conductance and thermovoltage were simultaneously determined as the slope and the offset in voltage, respectively, of the *IV* curves. The Seebeck coefficient (*S*) is the ratio between the mean thermovoltage value obtained for each temperature difference. Figure 3 shows the linear fitting of the temperature difference dependence of the mean thermovoltage values obtained for the MeS···SMe contacted junctions (main conductance class) of each compound. The conductance class was determined by applying the k-means clustering algorithm to the conductance-distance traces from which the *IV* curves were recorded, to ensure the correlation between the thermopower data and the conductance histograms. Each of the compounds had a negative Seebeck coefficient, indicating that the conductance is LUMO dominated. Previous studies with SMe anchors provide precedent for either HOMO- or LUMO-dominated transport, depending on the molecular backbone.^46−52^ and a related cyclic thioether anchor gave HOMO-dominated transport.^53^ It is interesting to observe that the magnitude of the Seebeck coefficients is unusually low (**L**^**p**^ is −0.4 μV/K and **L**^**m**^ is −0.5 μV/K), especially when compared to a cyclic thioether-terminated OPE3 derivative (1,4-bis((2,3-dihydrobenzo[*b*]thiophen-5-yl)ethynyl)benzene (*S* = −11.4 μV/K),^54^ meaning that in the present molecules the Fermi energy is close to the middle of the HOMO–LUMO. Upon coordination to palladium, the magnitude of the Seebeck coefficients modestly increased relative to the ligands for both **Pd**^**p**^ (−0.7 μV/K) and **Pd**^**m**^ (−1.3 μV/K) the *meta* compounds (**Pd**^**m**^ and **L**^**m**^) having a Δ*H*–L of 0.33 eV, while the *para* compounds (**Pd**^**p**^ and **L**^**p**^) have a Δ|H–L| of 0.05 eV. This suggests that while the H–L contraction plays a role in increasing the value of *S*, the shift of the resonance relative to the Fermi energy (*E*_F_) plays a greater role, as Blankevoort et al. concluded in a recent study of other molecules with low H-L gaps.^46^

### Theoretical Simulations

**Figure 4:**
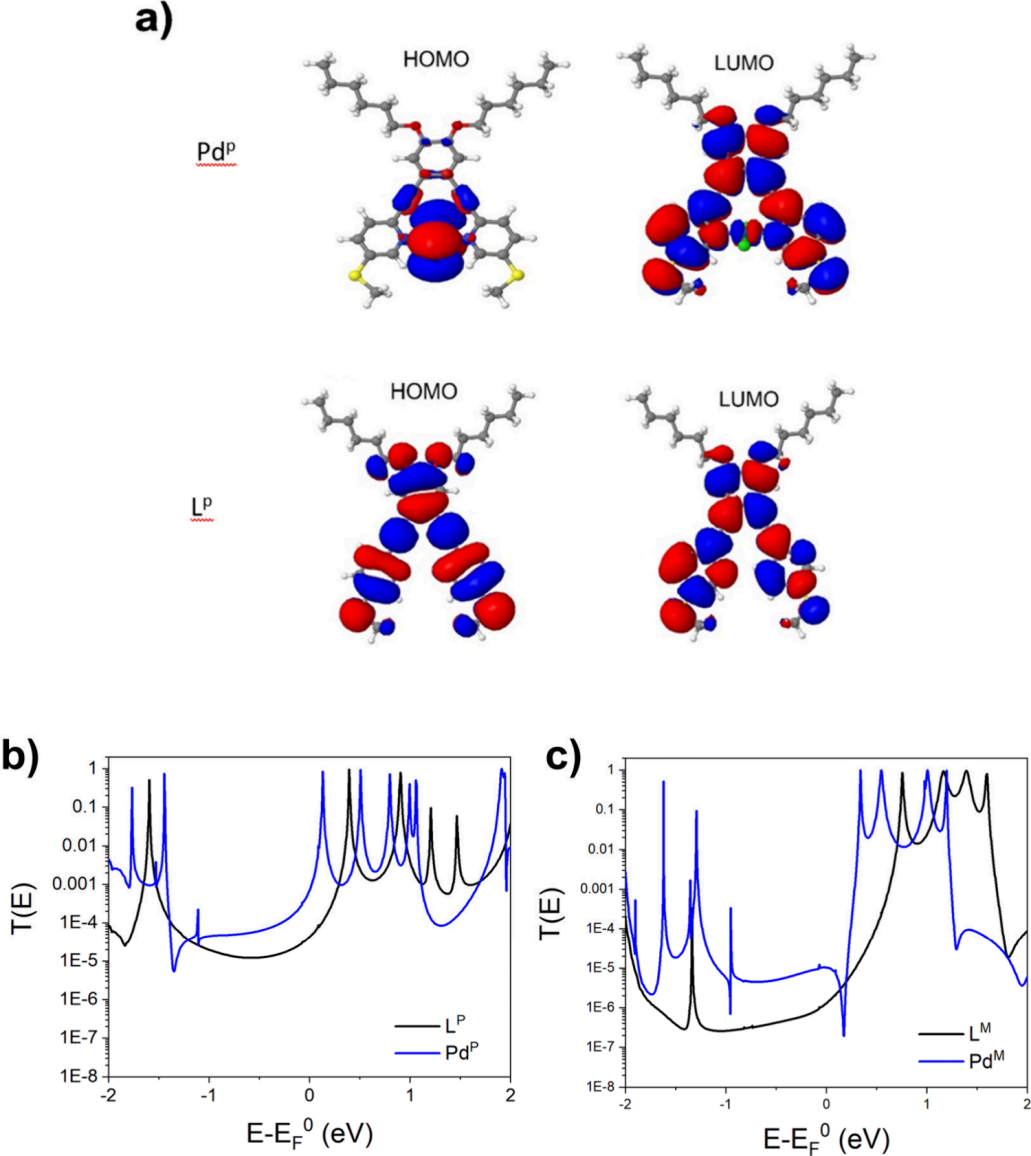
a) HOMO and LUMO orbitals of **Pd**^**p**^ and **L**^**p**^. b) Zero bias transmission coefficient T(E) for molecules **L**^**p**^ and **Pd**^**p**^ and c) molecules **L**^**m**^ and **Pd**^**m**^ contacted to gold electrodes via the SMe anchor groups. E_F_^0^ is the DFT-predicted Fermi energy.

The electrical conductance and Seebeck coefficient of the molecules were investigated using a combination of the density functional code SIESTA^55^ and the quantum transport code GOLLUM^56^ (full details of the theoretical approach can be found in the SI). These methods have been used over the past two decades to predict the effect on transport properties of a range molecular features, including conformation,^57^ pendent groups,^58^ heteroatoms^59^ and molecular-scale quantum interference.^60^ For each studied molecule, the optimum geometry was first calculated. For the ligands, **L**^**p**^ and **L**^**m**^, the free rotation of the pyridyl groups about the acetylene bond gave rise to a range of local minima associated with different possible rotamers (see Figures S45 and S46). However, the rotation was halted upon metal coordination. As examples, a comparison of the frontier orbitals of the *para*-connected molecules, **L**^**p**^ and **Pd**^**p**^, is shown in Figure 4a (see Figure S48 for frontier orbitals of other molecules). Here we can observe that the HOMO and LUMO of the ligand **L**^**p**^ are delocalized over the entire conjugated portion of the molecule. However, for the metal complex, **Pd**^**p**^, the HOMO is localized to the metal center while the LUMO remains delocalized over the conjugated portion of the ligand, typical of imine-based coordination complexes. Similar behavior was observed for the *meta* compounds **L**^**m**^ and **Pd**^**m**^ (Figure S49).

**Figure 5:**
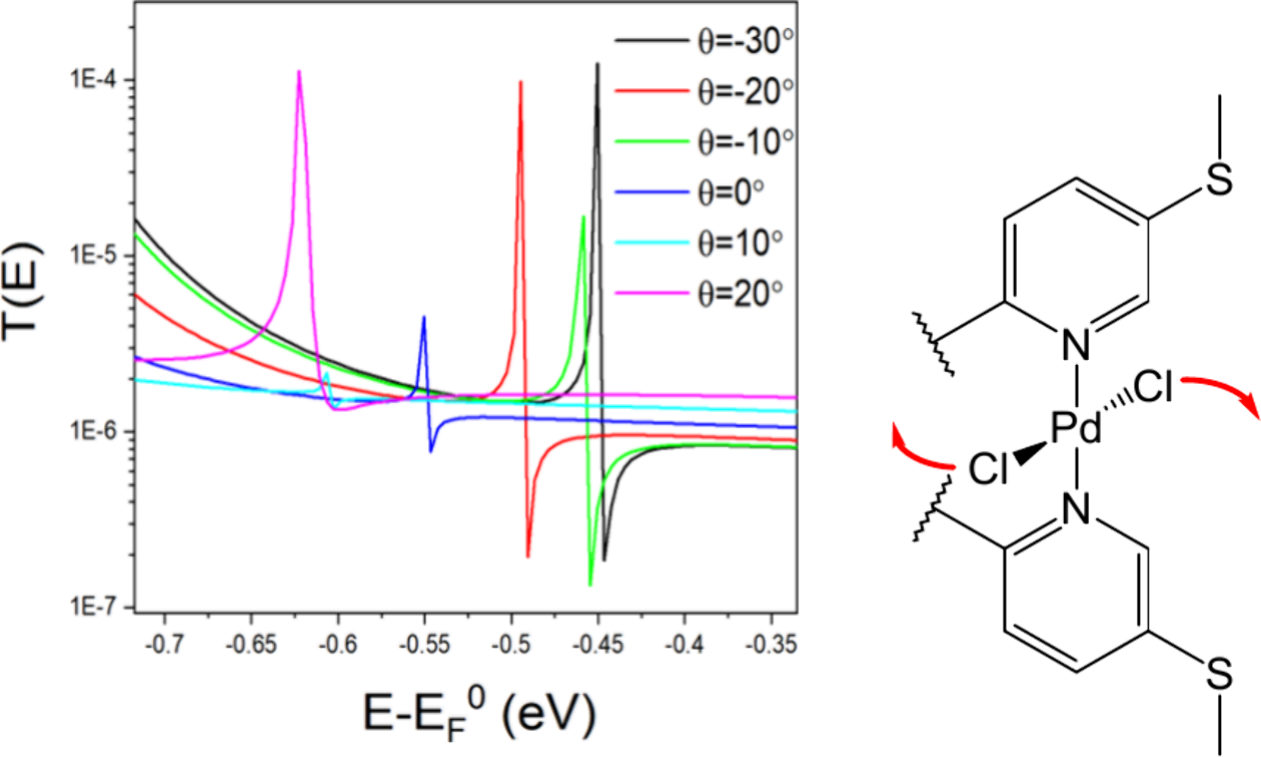
T(E) against energy E for the dihedral angle θ about the equilibrium position 0° in **Pd**^**p**^.

The molecules were then attached to gold electrodes via the sulfur of the thiomethyl anchor groups in the optimum binding position (Figures S52 and S53) and the transmission coefficient T(E) was calculated. Figures 4b and 4c present a comparison of the *para*-connected and *meta*-connected molecules. The conductance of **L**^**m**^ is consistently lower than that of **L**^**p**^ because the break in conjugation results from the thiomethyl anchor in the *meta* position. Upon coordination with the metal, the LUMO is stabilized moving the resonance closer to the DFT calculated Fermi energy (E_F_^0^); this occurs more significantly for **Pd**^**p**^ than **Pd**^**m**^ which accounts for the greater conductance enhancement for **Pd**^**m**^, similar to observations made by Ponce et al.^12^ Two new features were observed in addition to the HOMO–LUMO contraction: (i) a DQI feature at E-E_F_^0^ = 0.15 eV for **Pd**^**m**^ that appears to play no active role in the conductance; and (ii) the addition of the localized HOMO orbital produces resonance with a Fano feature (E-E_F_^0^ = −1 eV) well away from the Fermi energy, similar to those observed by Chelli et al. for 2,2′-bipyridine-based metal complexes.^13^ Interestingly, the Fano resonance shifts by almost 0.2 eV with a 50° rotation about the N–Pd–N axis for both **Pd**^**p**^ and **Pd**^**m**^ due to the *trans*-PdCl_2_ ability to rotate in a pendulum fashion (Figure 5 for **Pd**^**p**^). Although this feature is not involved in the molecular conductance, it demonstrates a previously unexplored means of manipulating a Fano resonance. The Pd atom does not produce a significant additional conductance pathway. As shown in Figure 4, transport is LUMO dominated, whereas, as shown in Figure S48, the presence of Pd atoms mainly affects the HOMO. Furthermore, the latter molecular orbital is localized on the Pd, with only a small weight on the SMe anchor groups and therefore, as shown in Figure 4, the associated transport resonance is extremely narrow.

From the DFT calculations it can be concluded that within the range of fluctuations, upon Pd coordination there is no significant difference between the shift of the LUMOs in the *meta* and *para* systems. For the *para* systems, Table S4 shows that for the various rotamers considered, the **L**^**p**^ LUMO varies between −2.54 eV and −3.00 eV, whereas Table S6 shows that the **Pd**^**p**^ LUMO varies between −2.96 eV and −3.34 eV. On the other hand, for the *para* systems, Table S5 shows that for the various rotamers considered, the **L**^**m**^ LUMO varies between −2.60 eV and −2.89 eV, whereas Table S7 shows that the **Pd**^**m**^ LUMO varies between −3.01 eV and −3.28 eV.

When evaluating the conductance and Seebeck coefficients, E_F_ = E_F_^0^ – 0.5 eV was chosen as this gave the closest approximation to the measured values and is consistent with the LUMO dominated conductance. However, due to the shallow gradient in the HOMO–LUMO gap it is difficult to accurately calculate the magnitude of the Seebeck coefficient (Table 2).

**Table 2:** Computed Conductance and Seebeck Coefficient Values^a^ for **L**^**p**^, **L**^**m**^, **Pd**^**p**^, and **Pd**^**m**^

molecule	conductance [log(*G*(E_F_)/*G*_0_]	*S*(*E*_F_) (μV/K)	conductance [log(*G*(*E*_F_^1^)/*G*_0_]	*S*(*E*_F_^1^) (μV/K)
**L**^**p**^	–4.65	–49.9	–5.21	–3.7
**L**^**m**^	–6.17	–38.1	–6.67	–9.5
**Pd**^**p**^	–2.82	–337.0	–4.51	–11.0
**Pd**^**m**^	–5.26	–84.1	–5.60	–8.4

a The conductance and Seebeck values evaluated at room temperature for two different Fermi energies. The original DFT calculated Fermi energy *E*_F_^0^ and *E*_F_^1^ = *E*_F_^0^ – 0.5 eV give better agreement with the experimental measured values.

## Conclusions

Two new 1,2-bis(2-pyridylethynyl)benzene (bpb)-based ligands and their *trans*-palladium dichloride complexes were synthesized with thiomethyl contact groups in either the *para* or *meta* positions of the pyridyl groups. Through a comparison of air/solvated conductance measurements and XPS data the stability of the complexes and ligands on a gold surface was assessed and subsequently a strategy that reduces undesired reactions with the surface was found. This highlights the importance of establishing by rigorous experiments what is actually being measured in a molecular junction. Although the free ligands **L**^**m**^ and **L**^**p**^ show the typical lower conductance for the *meta* isomer compared to the *para* isomer, upon coordination with palladium this difference is suppressed, which can be attributed to the contraction of the H–L energy gap being more significant for the *meta* compounds (**Pd**^**m**^ and **L**^**m**^). Additionally, coordinating a metal ion results in a modest increase in the magnitude of the Seebeck coefficient, which does not appear to be exclusively related to the H–L contraction. Instead, it is dependent upon how the resonances shift relative to E_F_, demonstrating that although metal coordination is a potential route for enhancing the Seebeck coefficient, in future work consideration must also be given to features that can influence the positions of the resonant features relative to the E_F_, such as judicious anchor group selection.

## Experimental Section

### Equipment

NMR spectra were recorded in deuterated solvent solutions on a Varian VNMRS-600 spectrometer and referenced against solvent resonances (^1^H, ^13^C). Accurate mass tandem mass spectrometer equipped with Atmospheric Pressure Gas Chromatography (APGC) and Atmospheric Solids Analysis Probe (ASAP) data were recorded on a high-resolution Xevo QTOF (Waters). For the differences quoted for *m*/*z* (|Δ*m*/*z*|) the value used is the mass error which is the difference between the experimental and theoretical mass values expressed in parts per million; this is calculated by subtracting the theoretical mass from the experimental values, this value is divided by the theoretical mass and multiplied by one million to convert to parts per million. This provides a means of comparing error independent of the ion’s mass. Microanalyses were performed by Elemental Analysis Service at Durham University, UK. UV–visible absorbance spectra of the compounds were recorded to provide an estimate of the HOMO–LUMO (H-L) energy gap for the theoretical calculations (see Figure S20, Table S2). The measurements were recorded in DCM solutions using an Evolution 220 Thermo Scientific spectrometer. XPS spectra were recorded on a Kratos AXIS ultra DLD spectrometer equipped with an Al Kα X-ray monochromatic source (1486.6 eV) and using 20 eV as pass energy.

### Molecular Conductance Measurements

The k-means clustering algorithm was used to separate the traces with similar conductance, leading to a single conductance peak in the histogram. The data was introduced with each trace as a linear vector, created by appending the raw data of a matrix constructed from the 2d-histogram with 40 bins between −1 and −6 log(G/G_0_) values and 30 bins between 0 and 2.5 nm of displacement. There was also appended another vector from a 1d-histogram with 100 bins between −1 and −6.5 log(G/G_0_) values. Then, a variable number of clusters, up to 6, were separated, some elucidating different conductance behaviors and some formed by tunnel traces. The clusters with a molecular feature are analyzed again to remove the remaining tunnel traces. All the clusters containing traces with molecular features were put together and the algorithm was applied again, since clusters describing similar conductance behavior may have been separated, as well as some traces may have been misclassified. The separation was performed now between the number of conductance classes expected by observing similar clusters obtained along the process.

## Data Availability

The data associated with this article are available in the manuscript and Supporting Information files.

## Supporting Information Available

NMR spectra, photophysical data, conductance and Seebeck measurements, XPS data and theoretical data, and crystallographic data for compound **Pd**^**p**^ (CSD 2345694) (PDF)
Crystallographic data for compound **Pd**^**p**^ (CSD 2345694) (CIF)

## Supplementary Material

ao4c06475_si_001.pdf

ao4c06475_si_002.cif
