# Supplementary material for: The Conductance and Thermopower Behavior of Pendent Trans-Coordinated Palladium(II) Complexes in Single-Molecule Junctions
Source: ACS Omega. 2024 Aug 28;9(36):38303–12. doi: 10.1021/acsomega.4c06475 (PMC11391538; doi:10.1021/acsomega.4c06475)
Supplement: Supplementary file 1 — ao4c06475_si_001.pdf [file ao4c06475_si_001.pdf]

SUPPORTING INFORMATION

The Conductance and Thermopower Behavior of Pendent *Trans*-Coordinated Palladium(II) Complexes in Single-Molecule Junctions

Pablo Bastante,[1] Ross J. Davidson,[2] Wafa Al Malki,[3] Rebecca J. Salthouse,[2] Pilar Cea,[4,5] Santiago Martin,[4,5] Andrei S. Batsanov,[3] Colin J. Lambert,[3] Martin R. Bryce,[2] Nicolas Agrait[1]

Affiliation:

[1]*Departamento de Física de la Materia Condensada C-III, and Instituto Universitario de Ciencia de Materiales "Nicolás Cabrera" , Universidad Autónoma de Madrid, E-28049 Madrid, Spain*

[2] *Department of Chemistry, University of Durham, Stockton Road, Durham, DH1 3LE, U.K.*

[3]*Department of Physics, University of Lancaster, Lancaster LA1 4YB, U.K.*

[4]*Instituto de Nanociencia y Materiales de Aragón (INMA), CSIC-Universidad de Zaragoza, 50009, Zaragoza, Spain*

[5]*Departamento de Química Física, Universidad de Zaragoza, 50009, Zaragoza, Spain and Laboratorio de Microscopias Avanzadas (LMA), Universidad de Zaragoza, 50018, Zaragoza, Spain*

Table of Contents

a) Experimental

1. Synthesis S3
2. NMR spectra of reported compounds S11
3. X-ray Crystallography S20

b) Photophysical measurements S23

c) Molecular conductance

1. Conductance peaks exploration S24
2. 2D Conductance-distance histograms S26
3. Junction length determination S32
4. Conductance switching S34

d) XPS results S37

e) Theoretical calculations S38

References S47

a) Experimental

1. Synthesis: General details. 2-Bromo-5-(methylthio)pyridine[1], 4-ethynylthioanisole[2], 2-((trimethylsilyl)ethynyl)pyridine[3] and 1,2-bis(hexyloxy)-4,5-diiodobenzene[4] were prepared according to published methods; all other chemicals were sourced from standard chemical suppliers.

2-bromo-4-(methylthio)pyridine

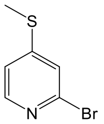

n-Butyllithium (2.5 M, 8.54 mL, 21.3 mmol) was added to a solution containing 2,4-dibromopyridine (5.00 g, 21.3 mmol) in Et_2O (70 mL) at -78 °C and stirred for 1 h before adding dimethyldisulfide (1.97 mL, 2.06 g, 22 mmol) and warming to room temperature, after which stirring was continued for 12 h before water was added to quench the reaction. DCM was added, the organic layer was collected and dried over $MgSO_4$ and filtered before the solvent was removed from the filtrate. The residue was purified using Kugelrohr distillation (120 °C, 0.1 mbar) to give a colorless oil. **Yield**: 3.91 (91%). **^{1}H NMR** (700 MHz; CD_2Cl_2): δ_H 8.10 (dd, $^3J_{HH}$ = 5.4 Hz, 1H, H_a), 7.27 (dd, $^4J_{HH}$ = 1.8 Hz, 1H, H_b), 7.07 (dd, $^3J_{HH}$ = 5.4 Hz, $^4J_{HH}$ = 1.7 Hz, 1H, H_c), 2.49 (s, 3H, H_d) ppm. **^{13}C{^{1}H} NMR** (125 MHz; $CDCl_3$): δ_C 153.4, 148.9, 142.2, 122.7, 119.2, 13.6 ppm. **Acc-MS**(ASAP$^+$): *m/z* 203.9475 $[M+H]^+$ calcd. for C_6H_7NSBr *m/z* 203.9483 (|Δ*m/z*| = 3.9 ppm). **Anal. Calc.** for C_6H_6BrNS: C, 35.31; H, 2.96; N, 6.86 %. **Found**: C, 35.56; H, 2.99; N, 6.80 %.

TMSA Sonogashira coupling: general method

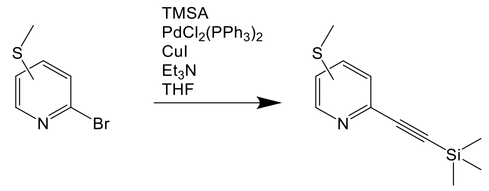

A solution of 2-bromo-5-(methylthio)pyridine or 2-bromo-4-(methylthio)pyridine (2.00 g, 9.80 mmol), $PdCl_2(PPh_3)_2$ (630 mg, 0.9 mmol), CuI (171 mg, 0.9 mmol), and Et_3N (5 mL) in THF (50 mL) was degassed by bubbling argon through, after which TMSA (1.40 mL, 980 mg, 10.00 mmol) was added. The solution was heated to reflux for 16 h before cooling and removing the solvent under vacuum, the residue was then eluted on a silica column with a solvent gradient from neat hexane to DCM:Hexane (1:1).

5-(methylthio-2-((trimethylsilyl)ethynyl)pyridine

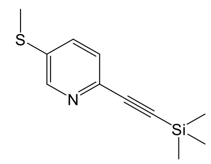

Yield: 3.55 g (70%). **^{1}H NMR** (600 MHz; CD_2Cl_2): δ_H 8.40 (d, $^3J_{HH}$ = 2.5 Hz, 1H, H_a), 7.48 (dd, $^3J_{HH}$ = 8.2 Hz, $^4J_{HH}$ = 2.4 Hz, 1H, H_b), 7.34 (d, $^3J_{HH}$ = 8.1 Hz, 1H, H_c), 2.51 (s, 3H, H_d), 0.26 (s, 9H, H_e) ppm. **^{13}C{^{1}H} NMR** (150 MHz; CD_2Cl_2): δ_C 147.3, 138.9, 135.9, 133.1, 126.9, 103.6, 94.3, 14.9, -0.65 ppm. **Acc-MS**(ASAP$^+$): *m/z* 205.1039 $[M+H]^+$ calcd. for $C_{13}H_{17}S$ *m/z* 205.1051 (|Δ*m/z*| = 5.9 ppm).

4-(methylthio)-2-((trimethylsilyl)ethynyl)pyridine

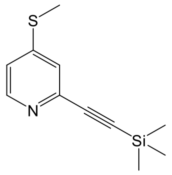

Yield: 2.04 g (63%). **^{1}H NMR** (600 MHz; CD_2Cl_2): δ_H 8.30 (d, $^3J_{HH}$ = 5.4 Hz, 1H, H_c), 7.25 (d, $^4J_{HH}$ = 1.9 Hz, 1H, H_a), 7.04 (dd, $^3J_{HH}$ = 5.4 Hz, $^4J_{HH}$ = 1.9 Hz, 1H, H_b), 2.49 (s, 3H, H_d), 0.27 (s, 9H, H_e) ppm. **^{13}C{^{1}H} NMR** (150 MHz; CD_2Cl_2): δ_C 150.6, 149.0, 142.5, 122.9, 119.2, 103.6, 94.2, 13.5, -0.6 ppm. **Acc-MS**(ASAP$^+$): *m/z* 222.0778 $[M+H]^+$ calcd. for $C_{11}H_{16}NSiS_2$ *m/z* 222.0773 (|Δ*m/z*| = 2.3 ppm).

Pyridyl-*ortho*-OPE: general synthesis

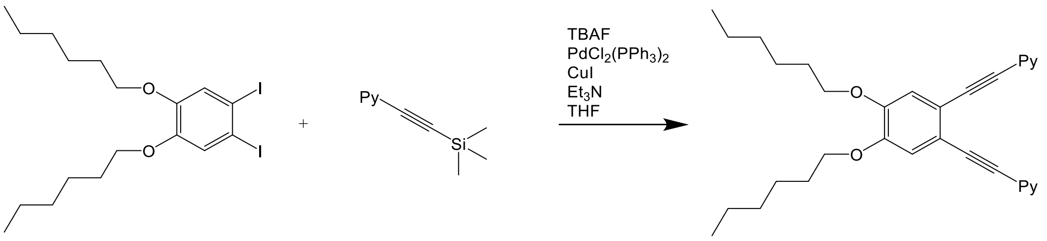

A solution of 2-((trimethylsilyl)ethynyl)pyridine (5-(methylthio)-2-((trimethylsilyl)ethynyl)pyridine, 4-(methylthio)-2-((trimethylsilyl)ethynyl)pyridine or 2-((trimethylsilyl)ethynyl)pyridine) (2.26 mmol), $PdCl_2(PPh_3)_2$ (140 mg, 0.2 mmol), CuI (38 mg, 0.2 mmol), 1,2-bis(hexyloxy)-4,5-diiodobenzene (0.58 g, 1.10 mmol), and Et_3N (2 mL) in THF (50 mL) was degassed by bubbling argon through, after which TBAF (1.0 M, 2.50 mL, 2.50 mmol) was added. The solution was heated to reflux for 16 h before cooling and removing the solvent under vacuum, the residue was then eluted on a silica column with a solvent gradient from neat DCM to DCM:ethyl acetate (9:1).

6,6'-((4,5-bis(hexyloxy)-1,2-phenylene)bis(ethyne-2,1-diyl))bis(3-(methylthio)pyridine) **L^p**

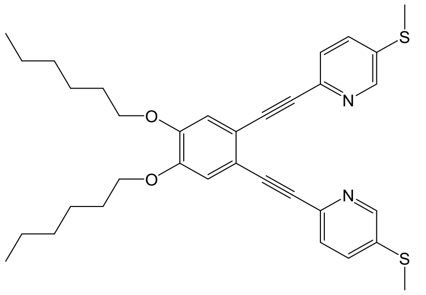

Yield: 0.20 g (45%). **^{1}H NMR** (600 MHz; CD_2Cl_2): δ_H 8.48 (s, 2H, H_a), 7.59 (d, $^3J_{HH}$ = 8.3 Hz, 2H, H_c), 7.54 (dd, $^3J_{HH}$ = 8.27 Hz, J = 2.40 Hz, 2H, H_b), 7.10 (s, 2H, H_d), 4.03 (t, $^3J_{HH}$ = 6.6 Hz, 4H, H_f), 2.55 (s, 6H, H_e), 1.84-1.80 (m, 4H, H_g), 1.50-1.46 (m, 4H, H_h), 1.38-1.35 (m, 8H, H_i+H_j), 0.93-0.91 (m, 6H, H_k) ppm. **^{13}C{^{1}H} NMR** (150 MHz; CD_2Cl_2): δ_C 149.7, 147.5, 139.5, 135.3, 133.3, 127.0, 117.9, 115.8, 91.5, 88.0, 69.2, 31.4, 29.0, 25.5, 22.5, 15.1, 13.7 ppm. **Acc-MS**(ASAP$^+$): *m/z* 573.2609 [M+H]$^+$ calcd. for $C_{34}H_{41}N_2O_2S_2$ *m/z* 573.2609 (|Δ*m/z*| = 0.0 ppm). **Anal. Calc.** for $C_{34}H_{40}N_2O_2S_2$: C, 71.29; H, 7.04; N, 4.89 %. **Found**: C, 71.07; H, 6.96; N, 4.62 %.

2,2'-((4,5-bis(hexyloxy)-1,2-phenylene)bis(ethyne-2,1-diyl))bis(4-(methylthio)pyridine) **L^m**

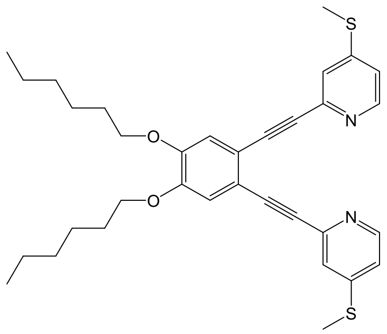

Yield: 0.10 g (30%). **^{1}H NMR** (600 MHz; CD_2Cl_2): δ_H 8.39 (d, $^3J_{HH}$ = 5.4 Hz, 2H, H_a), 7.54 (d, $^4J_{HH}$ = 1.9 Hz, 2H, H_c), 7.12 (s, 2H, H_d), 7.06 (dd, $^3J_{HH}$ = 5.4 Hz, $^4J_{HH}$ = 2.0 Hz, 2H, H_b), 4.04 (t, $^3J_{HH}$ = 6.6 Hz, 4H, H_f), 2.49 (s, 6H, H_e), 1.85-1.80 (m, 4H, H_g), 1.51-1.46 (m, 4H, H_h), 1.39-1.34 (m, 8H, H_i+H_j), 0.94-0.90 (m, 6H, H_k) ppm. **^{13}C{^{1}H} NMR** (150 MHz;

CD_2Cl_2): δ_C 150.17, 142.43, 136.64, 127.48, 123.39, 117.94, 115.59, 91.10, 69.28, 31.48, 28.98, 25.57, 22.56, 13.74 ppm. **Acc-MS**(ASAP$^+$): *m/z* 573.2611 $[M+H]^+$ calcd. for $C_{34}H_{41}N_2O_2S_2$ *m/z* 573.2609 (|Δ*m/z*| = 0.3 ppm). **Anal. Calc.** for $C_{34}H_{40}N_2O_2S_2{\cdot}\frac{1}{4}CH_2Cl_2$: C, 69.25; H, 6.87; N, 4.72 %. **Found**: C, 69.60; H, 6.79; N, 4.65 %.

2,2'-((4,5-bis(hexyloxy)-1,2-phenylene)bis(ethyne-2,1-diyl))dipyridine **L^{py}**.

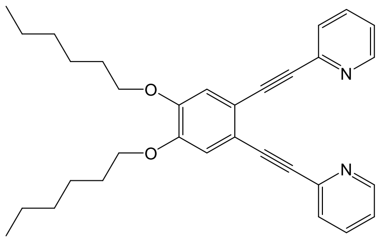

Yield: 134 mg (35%). **^{1}H NMR** (600 MHz, CD_2Cl_2) δ 8.71 (d, $^3J_{HH}$ = 5.2 Hz, 2H, H_a), 7.75 (td, $^3J_{HH}$ = 7.7 Hz, $^4J_{HH}$ =1.7 Hz, 2H, H_c), 7.64 (dt, $^3J_{HH}$ = 8.0 Hz, $^4J_{HH}$ = 1.1 Hz, 2H, H_d), 7.34 (ddd, $^3J_{HH}$ = 7.6 Hz, $^3J_{HH}$ = 5.1 Hz, $^4J_{HH}$ = 1.3 Hz, 2H, H_b), 7.13 (s, 2H, H_e), 4.05 (t, $^3J_{HH}$ = 6.6 Hz, 4H, H_f), 1.86 – 1.81 (m, 4H, H_g), 1.50 (p, $^3J_{HH}$ = 7.2 Hz, 4H, H_h), 1.39 – 1.35 (m, 8H, H_{j+i}), 0.94 – 0.91 (m, 6H, H_k) ppm. **^{13}C NMR** (150 MHz, CD_2Cl_2) δ 150.2, 150.1, 142.4, 136.6, 127.4, 123.4, 117.9, 115.6, 91.1, 69.3, 31.5, 29.0, 25.6, 22.6, 13.7 ppm. **HRMS** (ASAP$^+$) *m/z* 481.2859 $[M+H]^+$, calc. for $C_{32}H_{37}N_2O_2$ m/z 481.2855 (|Δ*m/z*| = 0.83 ppm). **Anal. Calc.** for $C_{32}H_{36}O_2N_2{\cdot}2CH_2Cl_2$: C, 62.78; H, 6.20; N, 4.31 %. **Found**: C, 63.97; H, 6.09; N, 4.42 %.

2,2'-((4,5-bis(hexyloxy)-1,2-phenylene)bis(ethyne-2,1-diyl))dipyridine synthesis

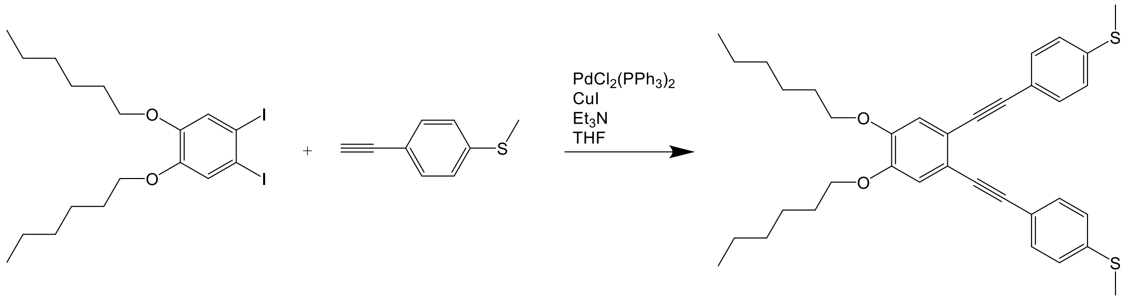

(((4,5-bis(hexyloxy)-1,2-phenylene)bis(ethyne-2,1-diyl))bis(4,1-phenylene))bis(methylsulfane) **SMe^p^**.

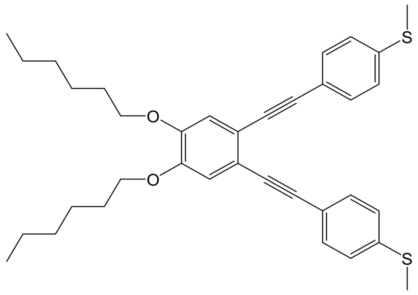

A solution containing $PdCl_2(PPh_3)_2$ (29 mg, 0.042 mmol), CuI (8 mg, 0.042 mmol), 1,2-bis(hexyloxy)-4,5-diiodobenzene (450 mg, 0.849 mmol), and Et_3N (10 mL) in THF (10 mL) was degassed by bubbling argon through, after which 4-ethynylthioanisole (252 mg, 1.69 mmol) was added. The solution was heated to reflux for 16 h before cooling and removing the solvent under vacuum, the residue was purified by two separate silica columns, first eluted a solvent gradient from neat hexane to hexane : ethyl acetate (9:1) followed by a second using a gradient from neat hexane to hexane : DCM (7:3). **Yield**: 213 mg (44%). **^{1}H NMR** (400 MHz, $CDCl_3$) δ 7.45 (d, $^3J_{HH}$ = 8.6 Hz, 4H, H_a), 7.19 (d, $^3J_{HH}$ = 8.6 Hz, 4H, H_b), 7.00 (s, 2H, H_c), 4.02 (t, $^3J_{HH}$ = 6.7 Hz, 4H, H_e), 2.50 (s, 6H, H_d), 1.84 (dt, $^2J_{HH}$ = 14.5, $^3J_{HH}$ = 6.7 Hz, 4H, H_f), 1.52 – 1.44 (m, 4H, H_g), 1.37 – 1.34 (m, 8H, H_{h+i}), 0.93 – 0.89 (m, 6H, H_j) ppm. **^{13}C NMR** (176 MHz, $CDCl_3$) δ 149.1, 139.1, 131.7, 125.9, 119.9, 118.6, 115.7, 91.8, 88.7, 69.1, 31.5, 29.1, 25.6, 22.6, 15.4, 14.0 ppm. **HRMS** (ASAP$^+$) *m/z* 571.2706 $[M+H]^+$, calc. for $C_{36}H_{43}O_2S_2$ m/z 571.2704 (|Δ*m/z*| = 0.35 ppm). **Anal. Calc.** for $C_{36}H_{42}O_2S_2$: C, 75.75; H, 7.42; N, 0. **Found**: C, 75.31; H, 7.10; N, −0.08.

Metal Complex Synthesis

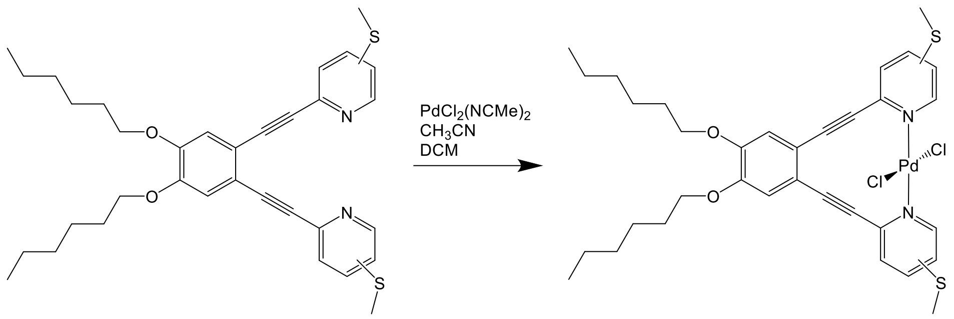

A solution of $PdCl_2(CH_3CN)_2$ (89 mg, 0.34 mmol) in acetonitrile (10 mL) was layered on to a solution of the ligand (200 mg, 0.34 mmol) in DCM. The layered solutions were left to stand at room temperature until a solid formed (approximately 2 days), after which time the solution was left to stand for an additional 2 days before the precipitate was collected by filtration.

Pd(L^p)Cl_2 **Pd^p**

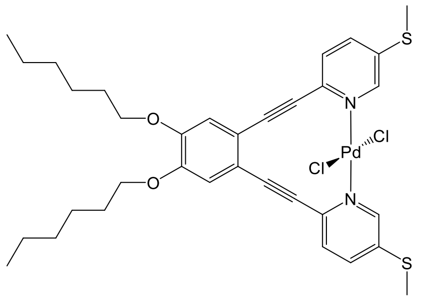

Yield: 0.15 g (62%). **^{1}H NMR** (600 MHz; CD_2Cl_2): δ_H ppm. 8.65 (d, $^4J_{HH}$ = 2.0 Hz, 2H, H_a), 7.60 (dd, $^3J_{HH}$ = 8.4 Hz, $^4J_{HH}$ = 2.2 Hz, 2H, H_b), 7.51 (d, $^3J_{HH}$ = 8.4 Hz, 2H, H_c), 7.21 (d, $^4J_{HH}$ = 1.0 Hz, 2H, H_d), 4.10 (t, $^3J_{HH}$ = 6.6 Hz, 4H, H_f), 2.62 (s, 6H, H_e), 1.86 (p, $^3J_{HH}$ = 6.8 Hz, 4H, H_g), 1.53-1.49 (m, 4H, H_h)[1], 1.39-1.36 (m, 8H, H_i+H_j), 0.93 (t, $^3J_{HH}$ = 7.4 Hz, 6H, H_k)

[1] Signal overlapping with water.

ppm. **^{13}C{^{1}H} NMR** (150 MHz; CD_2Cl_2): δ_C 150.6, 149.2, 140.7, 134.9, 127.9, 117.8, 115.8, 96.0, 89.8, 80.0, 69.3, 31.5, 28.9, 25.5, 22.5, 15.1, 13.7 ppm. **MS**(ASAP$^+$): *m/z* 749.101 $[M]^+$. **Anal. Calc.** for $C_{34}H_{40}Cl_2N_2O_2PdS_2\cdot½H_2O$: C, 53.79; H, 5.44; N, 3.69 %. **Found**: C, 53.60; H, 5.25; N, 3.45 %.

Pd(L^m)Cl$_2$ **Pd^m**

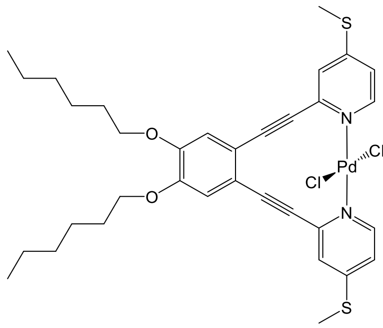

Yield: 0.11 g (44%). **^{1}H NMR** (600 MHz; CD_2Cl_2): δ_H 8.49 (dd, $^3J_{HH}$ = 6.3 Hz, 2H, H_a), 7.40 (d, $^3J_{HH}$ = 7.4 Hz, 2H, H_c), 7.22 (s, 2H, H_d), 7.12 (dd, $^3J_{HH}$ = 6.3 Hz, $^4J_{HH}$ = 2.3 Hz, 2H, H_b), 4.10 (t, $^3J_{HH}$ = 6.6 Hz, 4H, H_f), 2.55 (s, 6H, H_e), 1.89-1.84 (m, 4H, H_g), 1.53-1.49 (m, 4H, H_k, solvent overlap), 1.41-1.36 (m, 8H, H_i+H_j), 0.94-0.92 (m, 6H, H_k) ppm. **^{13}C{^{1}H} NMR** (150 MHz; CD_2Cl_2): δ_C 154.5, 150.9, 150.6, 143.5, 122.8, 119.9, 117.9, 116.0, 95.5, 89.7, 69.3, 31.5, 28.9, 25.5, 22.5, 13.9, 13.7 ppm. **MS**(ASAP$^+$): *m/z* 749.099 $[M]^+$. **Anal. Calc.** for $C_{34}H_{40}Cl_2N_2O_2PdS_2$: C, 54.44; H, 5.37; N, 3.73 %. **Found**: C, 54.24; H, 5.28; N, 3.61 %.

2. NMR spectra

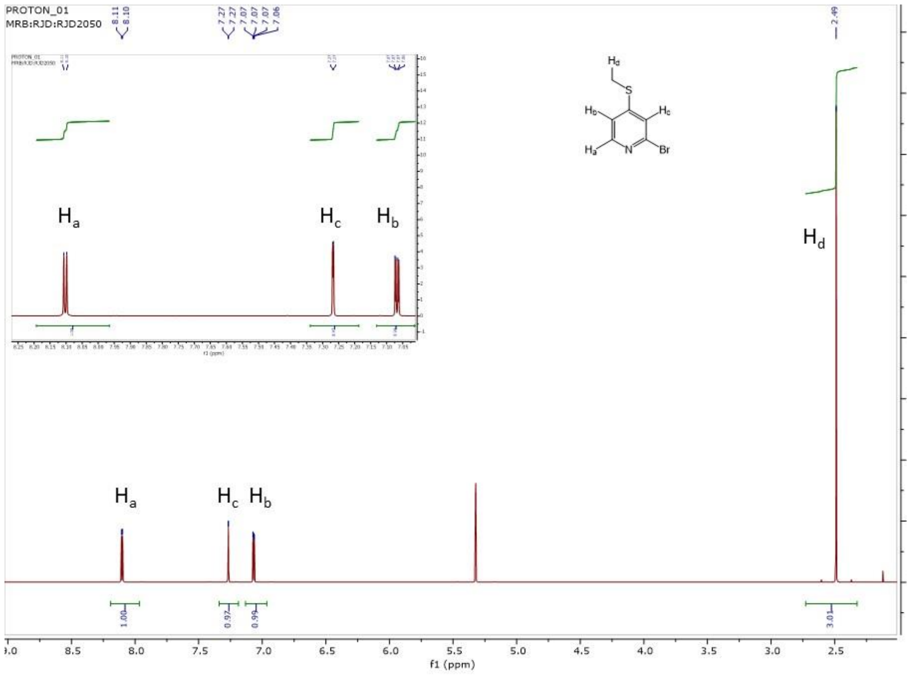

Figure S1. ^{1}H NMR spectrum of 2-bromo-4-(methylthio)pyridine recorded in CD_2Cl_2.

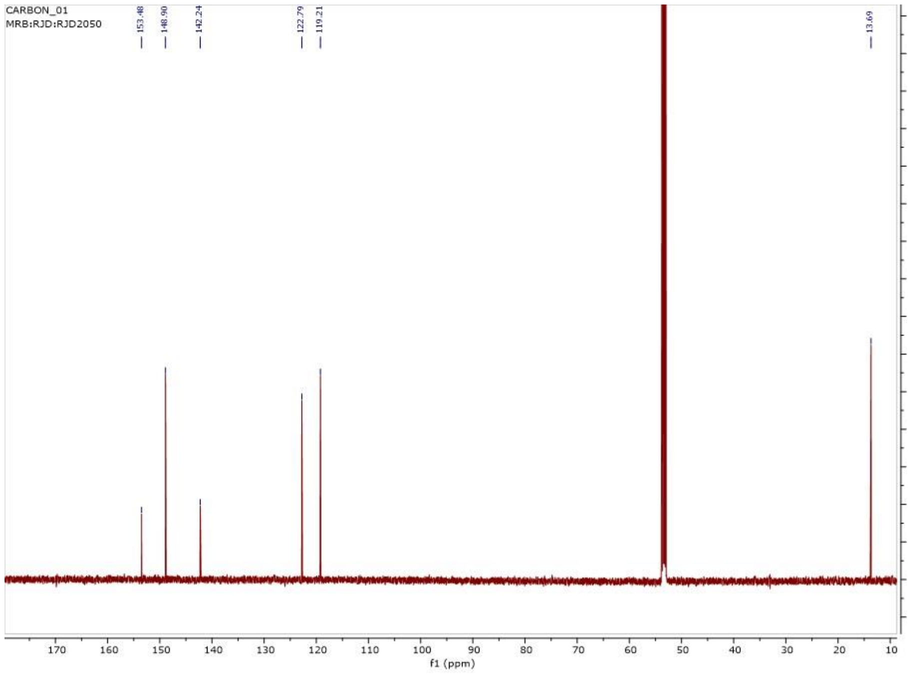

Figure S2. ^{13}C{^{1}H} NMR spectrum of 2-bromo-4-(methylthio)pyridine recorded in CD_2Cl_2.

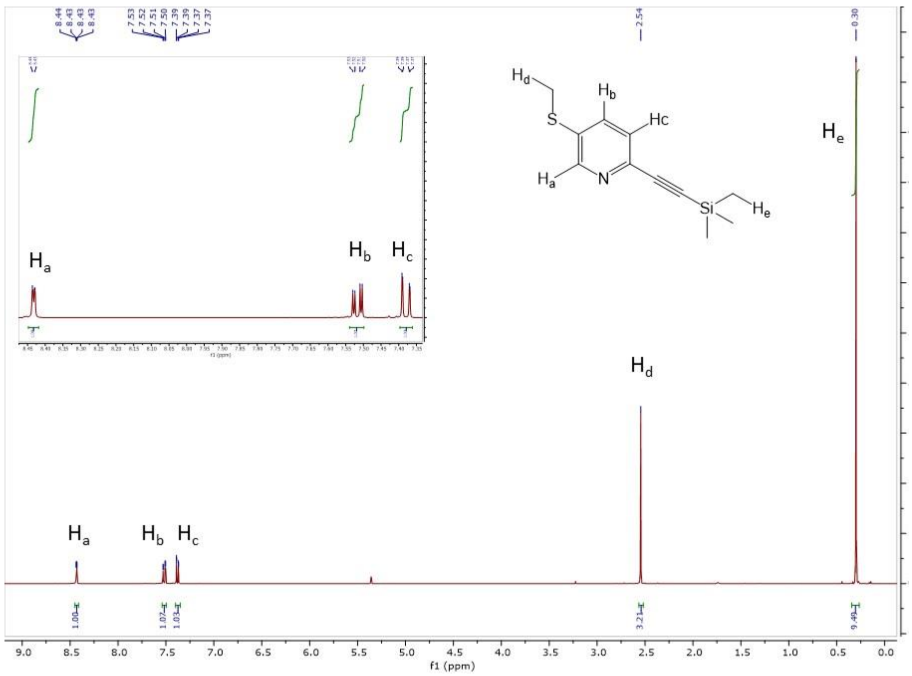

Figure S3. ^{1}H NMR spectrum of 5-(methylthio-2-((trimethylsilyl)ethynyl)pyridine recorded in CD_2Cl_2.

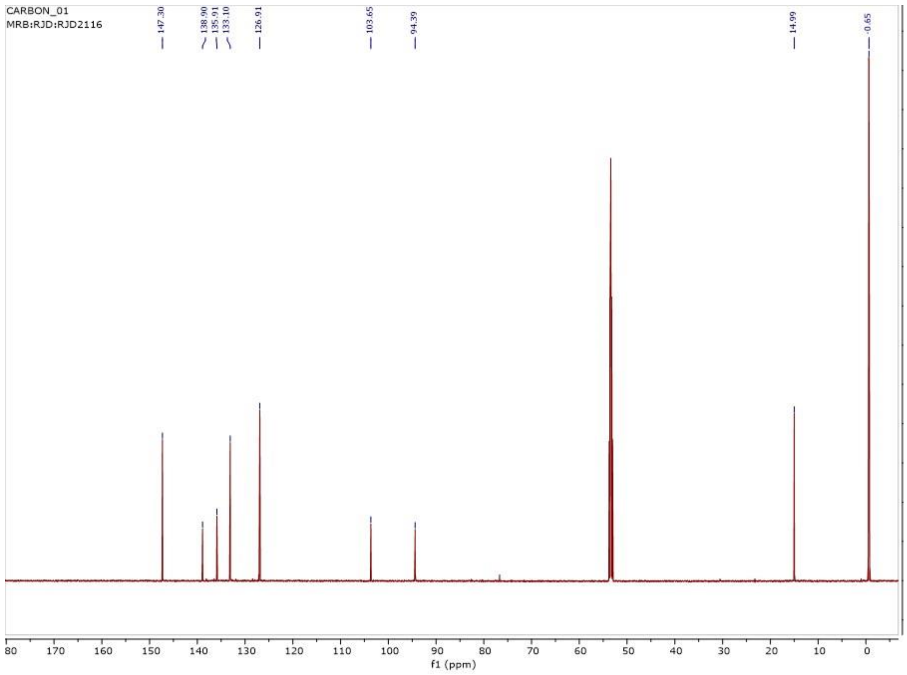

Figure S4. $^{13}C\{^{1}H\}$ NMR spectrum of 5-(methylthio-2-((trimethylsilyl)ethynyl)pyridine recorded in CD_2Cl_2.

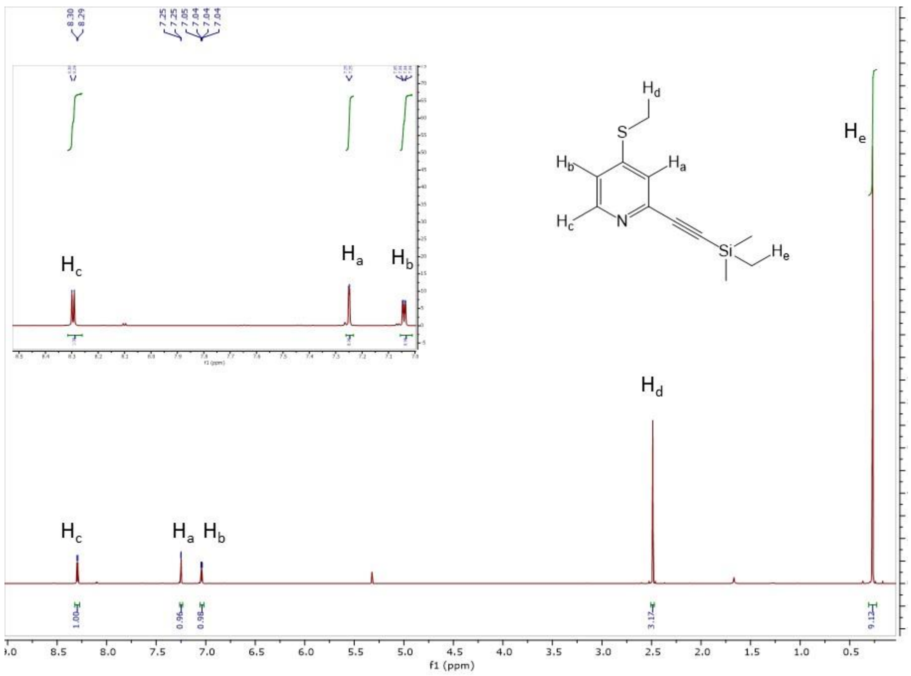

Figure S5. ^{1}H NMR spectrum of 4-(methylthio)-2-((trimethylsilyl)ethynyl)pyridine recorded in CD_2Cl_2.

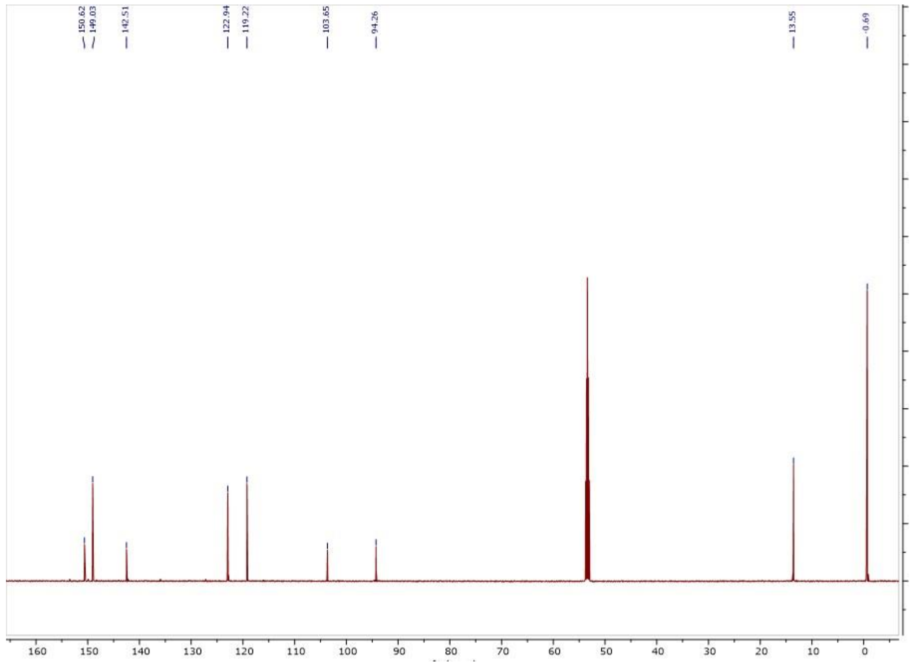

Figure S6. $^{13}C\{^{1}H\}$ NMR spectrum of 4-(methylthio)-2-((trimethylsilyl)ethynyl)pyridine recorded in CD_2Cl_2.

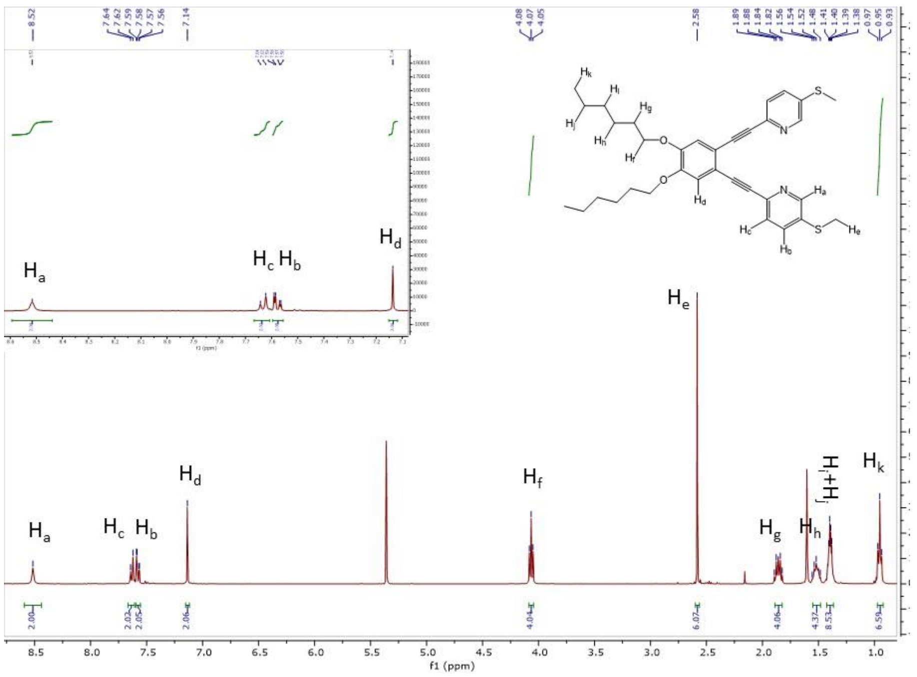

Figure S7. ^{1}H NMR spectrum of **L^p** recorded in CD_2Cl_2.

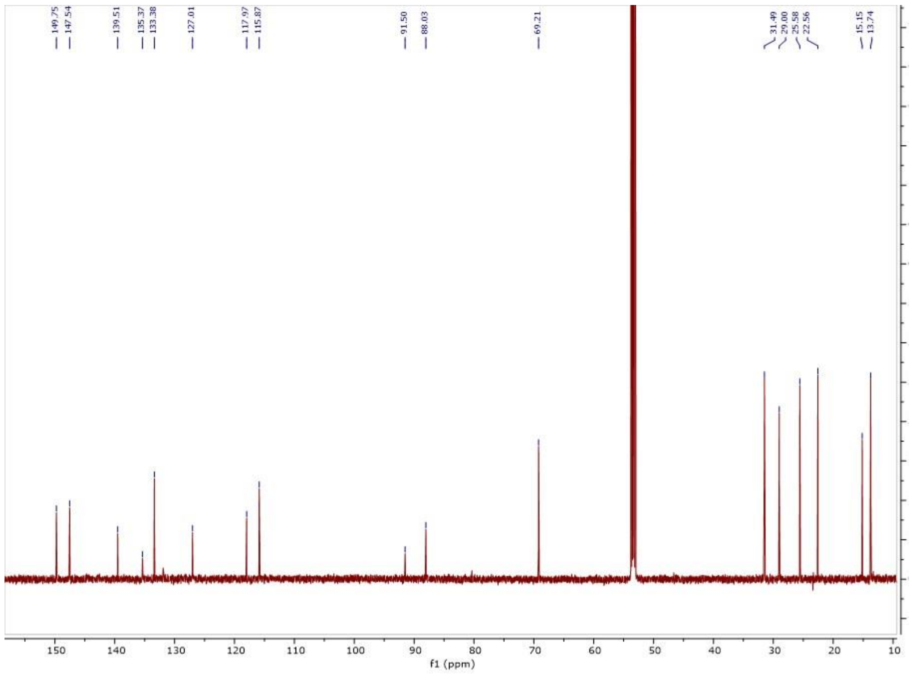

Figure S8. $^{13}C\{^{1}H\}$ NMR spectrum of **L^p** recorded in CD_2Cl_2.

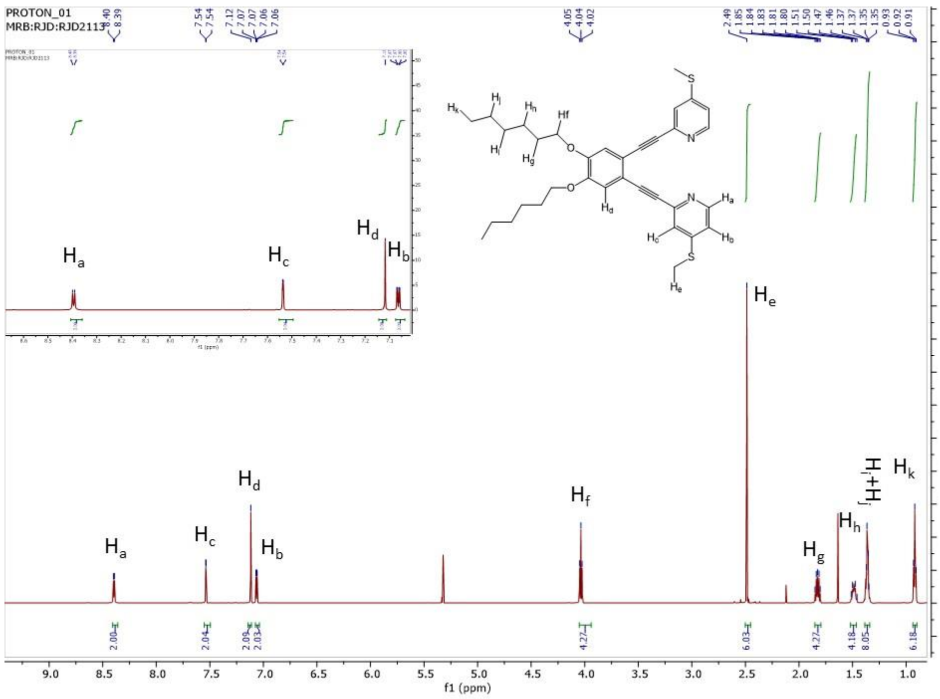

Figure S9. ^{1}H NMR spectrum of **L^m** recorded in CD_2Cl_2.

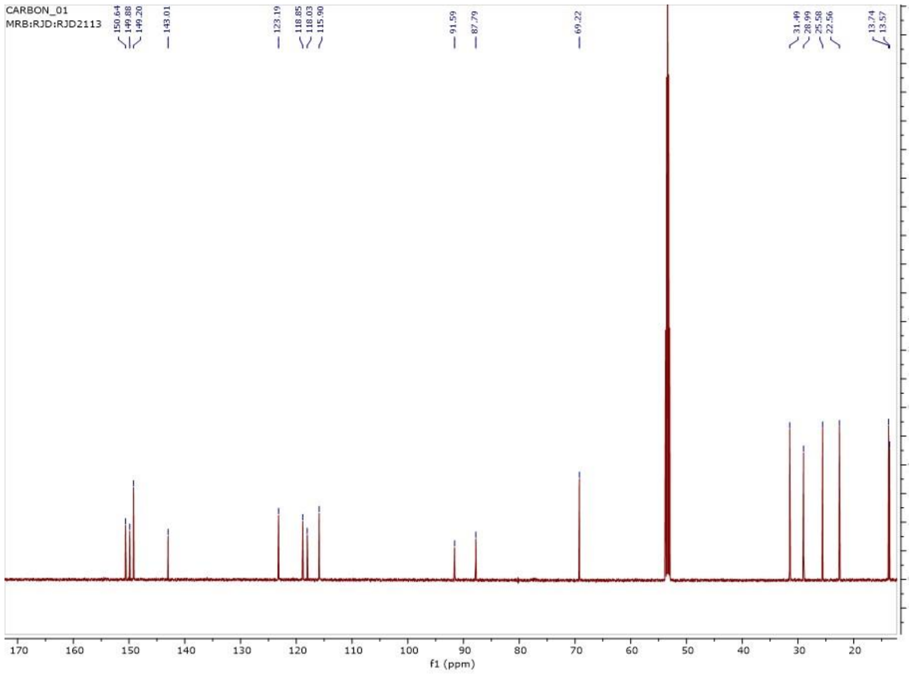

Figure S10. $^{13}C\{^{1}H\}$ NMR spectrum of **L^m** recorded in CD_2Cl_2.

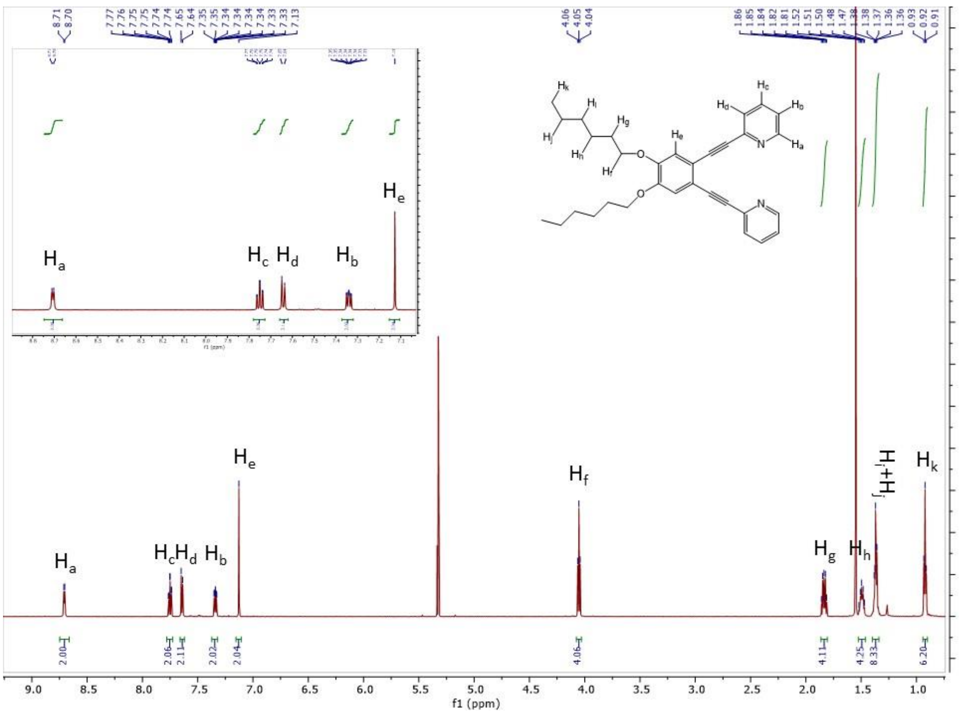

Figure S11. ^{1}H NMR spectrum of **L^{py}** recorded in CD_2Cl_2.

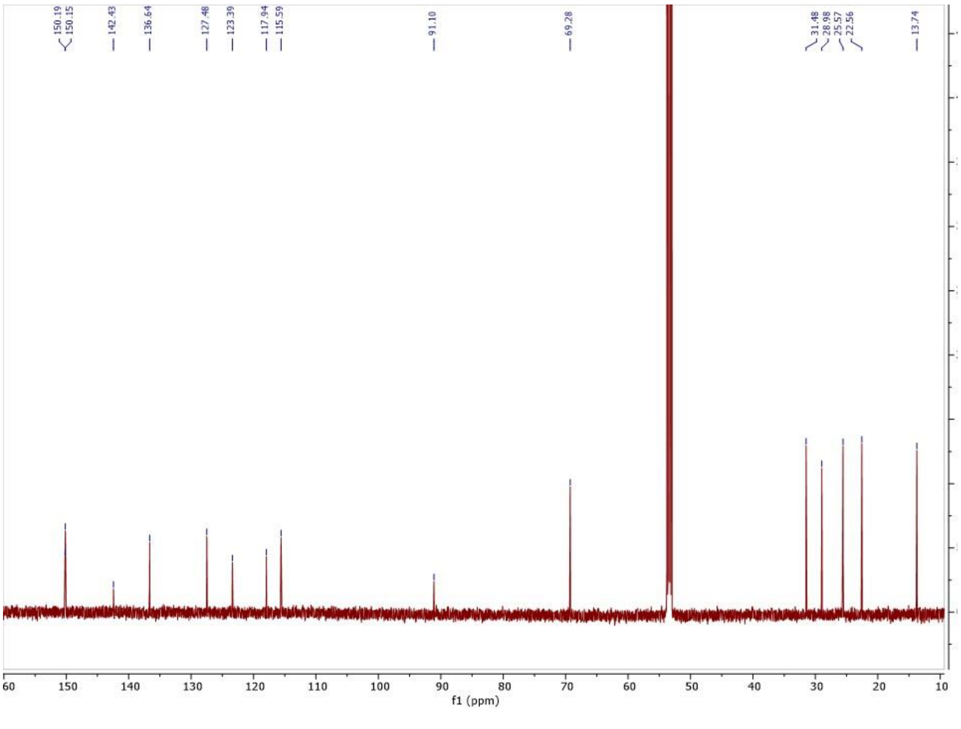

Figure S12. ^{13}C{^{1}H} NMR spectrum of **L^{py}** recorded in CD_2Cl_2.

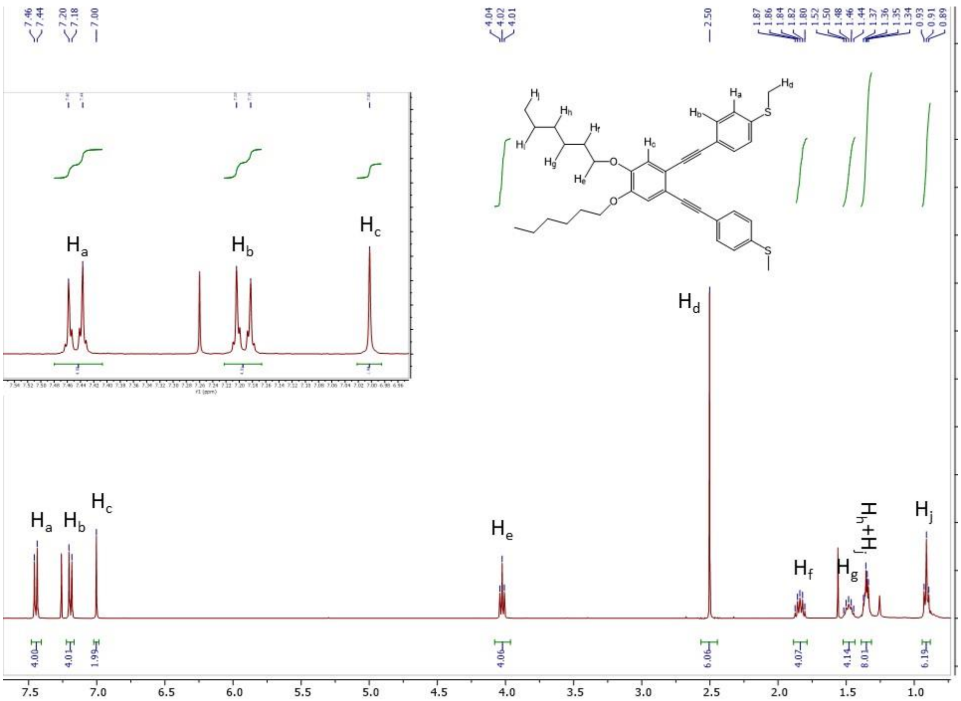

Figure S13. ^{1}H NMR spectrum of **SMe^{p}** recorded in $CDCl_3$.

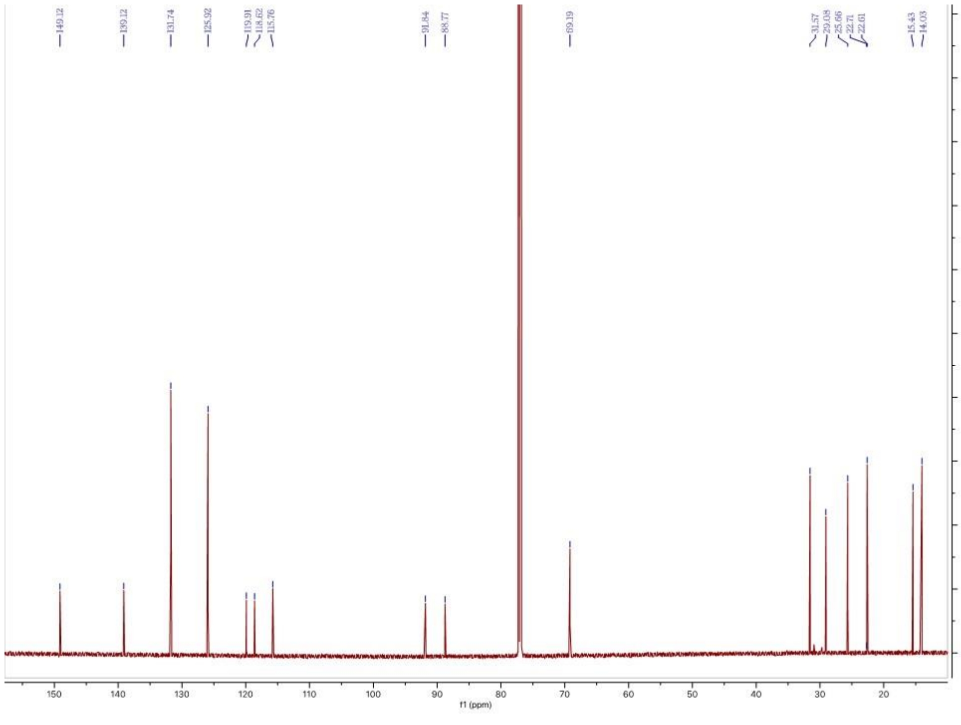

Figure S14. ^{13}C{^{1}H} NMR spectrum of **SMe^{p}** recorded in $CDCl_3$.

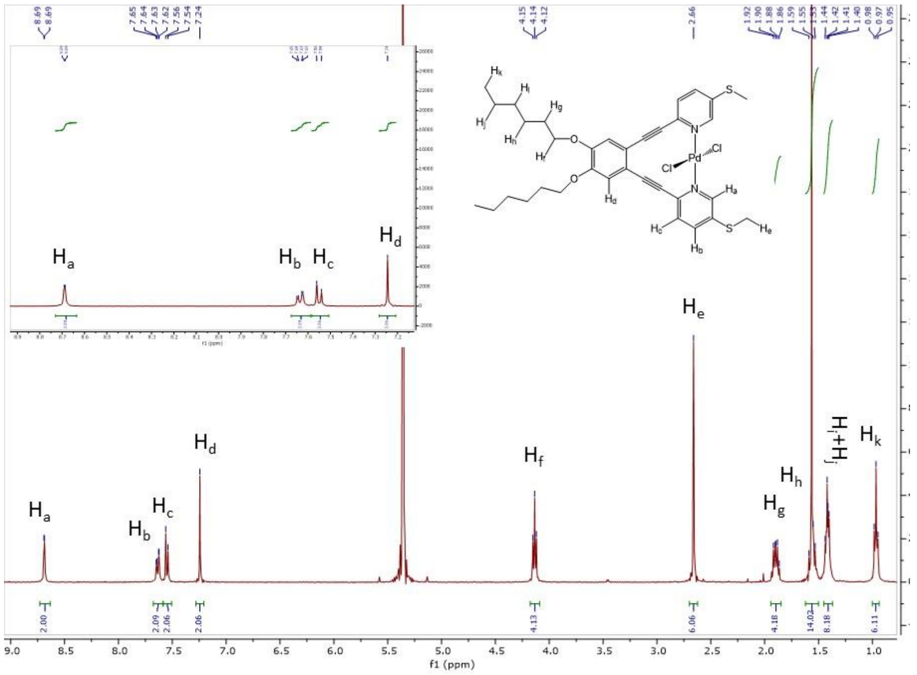

Figure S15. ^{1}H NMR spectrum of **Pd^p** recorded in CD_2Cl_2.

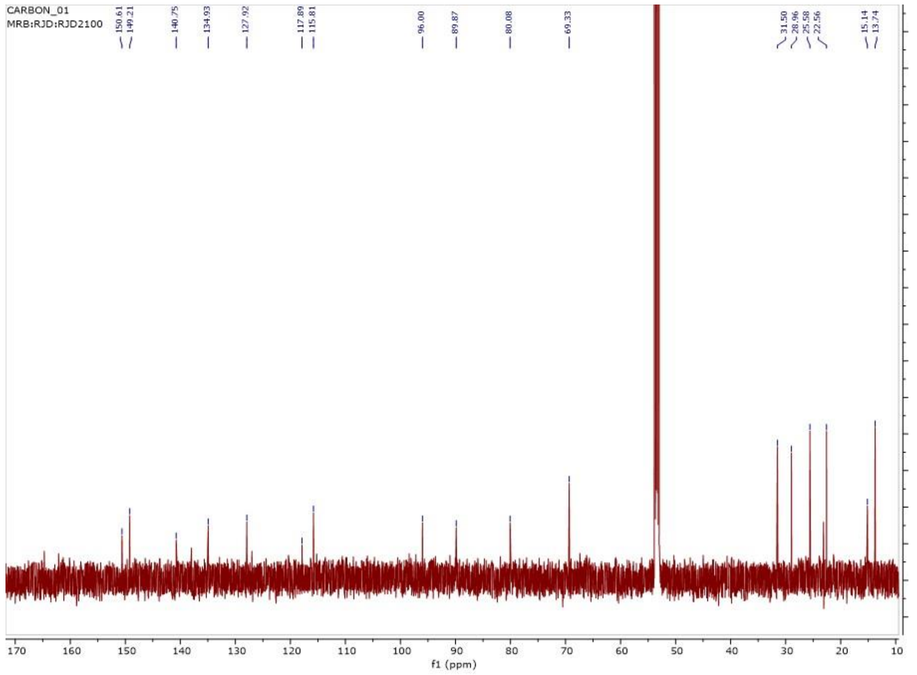

Figure S16. $^{13}C\{^{1}H\}$ NMR spectrum of **Pd^p** recorded in CD_2Cl_2.

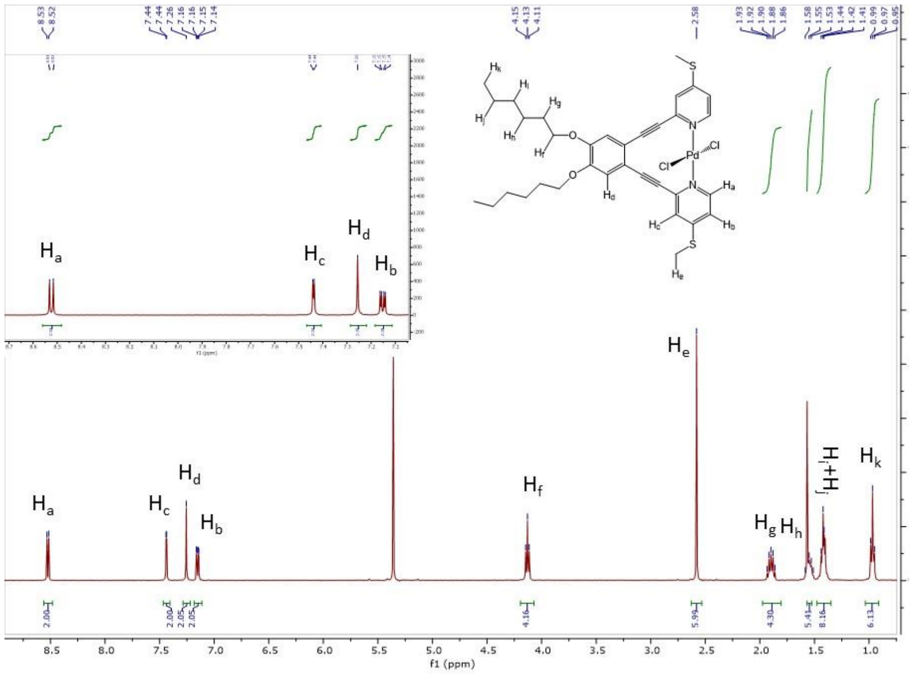

Figure S17. ^{1}H NMR spectrum of **Pd^m^** recorded in CD_2Cl_2.

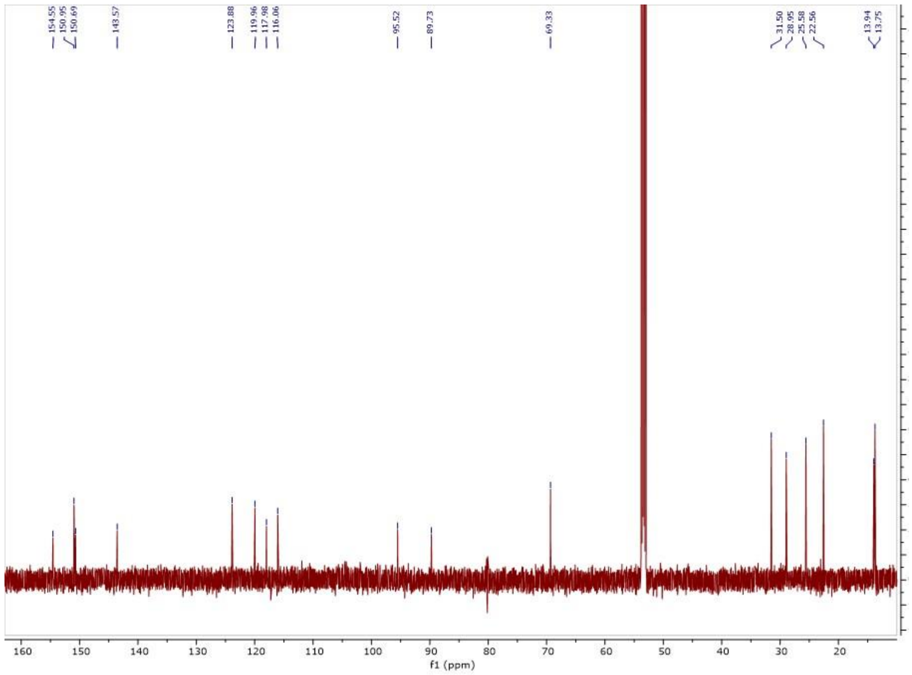

Figure S18. ${}^{13}C\{{}^{1}H\}$ NMR spectrum of **Pd^m^** recorded in CD_2Cl_2.

3. X-ray Crystallography

X-ray single crystal data for **Pd^p**•CH_2Cl_2 were collected on a Bruker D8 Venture diffractometer with a Photon III C14 MM CPAD detector, using λMo-*K*α radiation (λ = 0.71073Å) from a IμS microsource with focusing mirrors and corrected for absorption by numerical integration.[5] The crystal was cooled with a Cryostream 700+ (Oxford Cryosystems) open-flow N_2 cryostat. The structure was solved by dual-space intrinsic phasing method[5] and refined by full-matrix least squares against F^2 of all data using SHELXL program[5] on Olex2-1.5 platform.[6] Crystal data and experimental details are listed in Table S1. Crystallographic data for the structure have been deposited with the Cambridge Crystallographic Data Centre as supplementary publication CCDC 2345694.

The **Pd^p** molecule (Figure S19) lies astride a crystallographic twofold axis, sharing its site with small admixtures of certain by-products. Thus, the substituent at C(9) is an overlay of SMe (96.2(1)%) and Br (3.8(1)%), probably due to incomplete conversion of 2,4-dibromopyridine into 2-bromo-4-(methylthio)pyridine (see above). The halogeno ligand is an overlay of Cl(1) (96.3(1)%) and iodine (3.7(1)%) atoms, the latter possibly originating from CuI used in the synthesis of **L^p**. NMR techniques and elemental analysis gave no evidence of the halogeno species which suggests the amount present in the bulk to be negligible and that it is over-represented in the single crystal that was found to diffract to a suitable level. The *n*-hexyl chain is intensely disordered: atoms C(1) to C(4) are disordered between positions A, B and C with the occupancies of 0.45, 0.40 and 0.15, respectively, C(5) is disordered between positions A and B with occupancies 0.6 and 0.4. The dichloromethane molecule of crystallization is also disordered, Cl(2) lying on a twofold axis, Cl(3) and the CH_2 group being disordered between two positions related by this axis. Atoms C(1) to C(3) (all

positions), C(4A) and C(4C) were refined in isotropic approximation, other non-hydrogen atoms in anisotropic approximation, hydrogen atoms in riding mode.

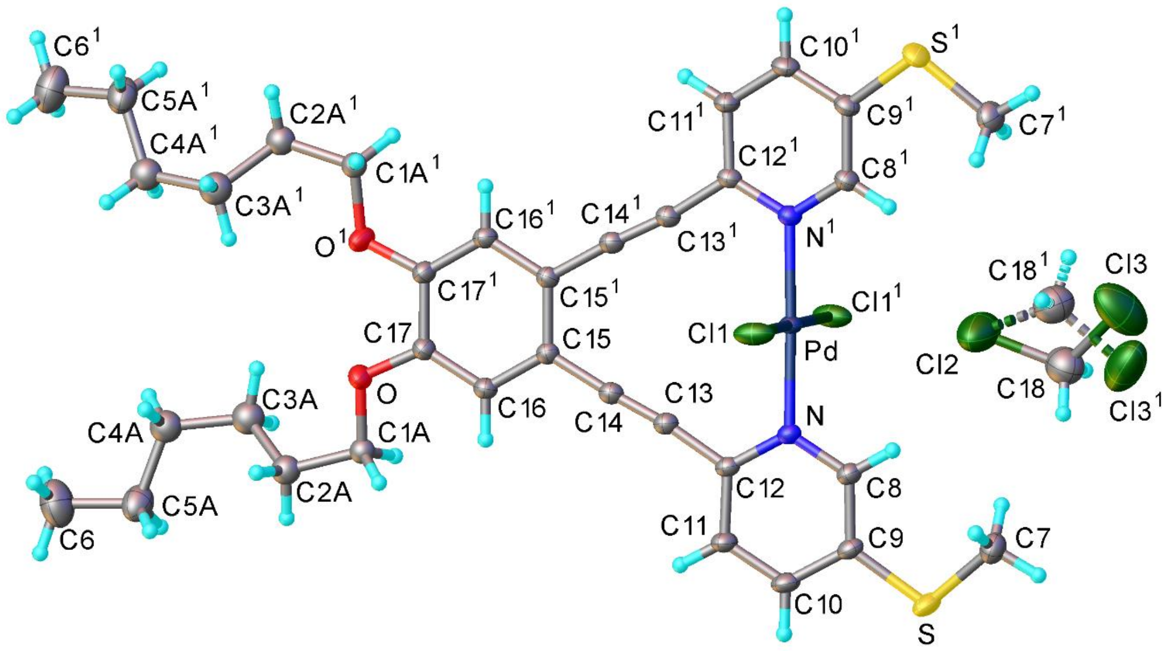

Figure S19. X-ray molecular structure of **Pd^p^**•CH_2Cl_2. Thermal ellipsoids are displayed at 50 % probability level, disorder removed for clarity. Atoms generated by twofold axis, are primed. Selected bond distances (Å): Pd-Cl 2.297(1), Pd-N 2.028(1), N-C(8) 1.347(2), N-C(12) 1.353(2), C(12-C(13) 1.418(2), C(13-C(14) 1.201(2), C(14)-C(15) 1.422(2).

Table S1. Crystal data and structure refinement for structures **Pd^p**.

Empirical formula (main component)	$C_{34}H_{40}Cl_2N_2O_2PdS_2 \cdot CH_2Cl_2$
Empirical formula (total)	$C_{33.93}H_{39.78}Br_{0.08}Cl_{1.93}I_{0.07}N_2O_2PdS_{1.92} \cdot CH_2Cl_2$
Formula weight	844.27
Temperature/K	150
Crystal system	Monoclinic
Space group	C2/c (No. 15)
a/Å	15.0954(11)
b/Å	21.8752(16)
c/Å	11.5083(8)
β/°	105.910(3)
Volume/Å^3	3654.6(5)
Z	4
ρ_{calc}, g/cm^3	1.534
μ/mm^{-1}	1.085
Reflections collected, unique, with I≥2σ(I)	57944, 8028, 7098
R_{int}	0.0365
Variable parameters /restraints/	249/81
Goodness-of-fit on F^2	1.087
Final R indexes [dat with I≥2σ(I)]	R_1 = 0.0382, wR_2 = 0.0904
Final R indexes [all data]	R_1 = 0.0453, wR_2 = 0.0940

b) Photophysical Measurements

A comparison of the free ligands ($\mathbf{L^p}$ and $\mathbf{L^m}$) shows that $\mathbf{L^p}$ has a significantly lower H-L energy gap than $\mathbf{L^m}$ attributed to the thiomethyl group in the *para* position extending the system's conjugation. Similar behavior was observed for the metal complexes except the H-L gaps were significantly smaller due to the metal coordination.

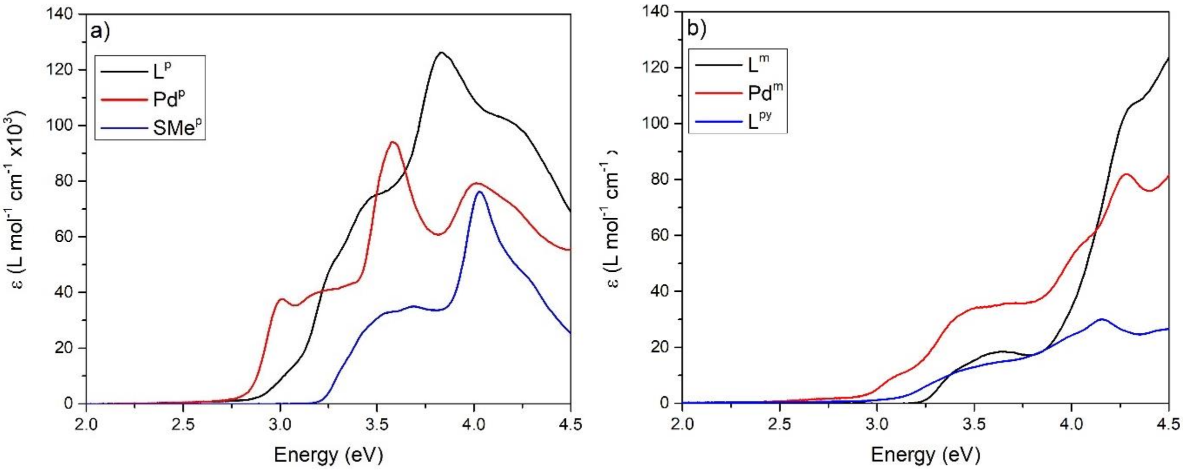

Figure S20. Electronic absorbance spectra of compounds a) $\mathbf{L^p}$, $\mathbf{Pd^p}$, $\mathbf{SMe^p}$; b) $\mathbf{L^m}$, $\mathbf{Pd^m}$ and $\mathbf{L^{py}}$.

Table S2. $\Delta\lvert(\text{HOMO-LUMO})\rvert$ energy for compounds $\mathbf{L^p}$, $\mathbf{Pd^p}$, $\mathbf{SMe^p}$, $\mathbf{L^m}$, $\mathbf{Pd^m}$, and $\mathbf{L^{py}}$.

Compound	$\Delta\lvert(\text{HOMO-LUMO})\rvert$ (eV)
$\mathbf{L^p}$	2.89
$\mathbf{L^m}$	3.24
$\mathbf{Pd^p}$	2.84
$\mathbf{Pd^m}$	2.91
$\mathbf{SMe^p}$	3.21
$\mathbf{L^{py}}$	3.08

c) Molecular Conductance

1. Conductance peaks exploration

Similar conductance values should be related to similar junction geometries. In solution, we would expect a reduced interaction of the molecules with the gold surface and between each other. Indeed, the conductance measurements in solution show that one of the peaks, the yellow one, became predominant, therefore it is assigned to the MeS…SMe contacted junction. The reference compounds served as a test to corroborate that the yellow peak is the connection with the SMe groups, where the connection to the N-groups was found to describe a lower conductance.

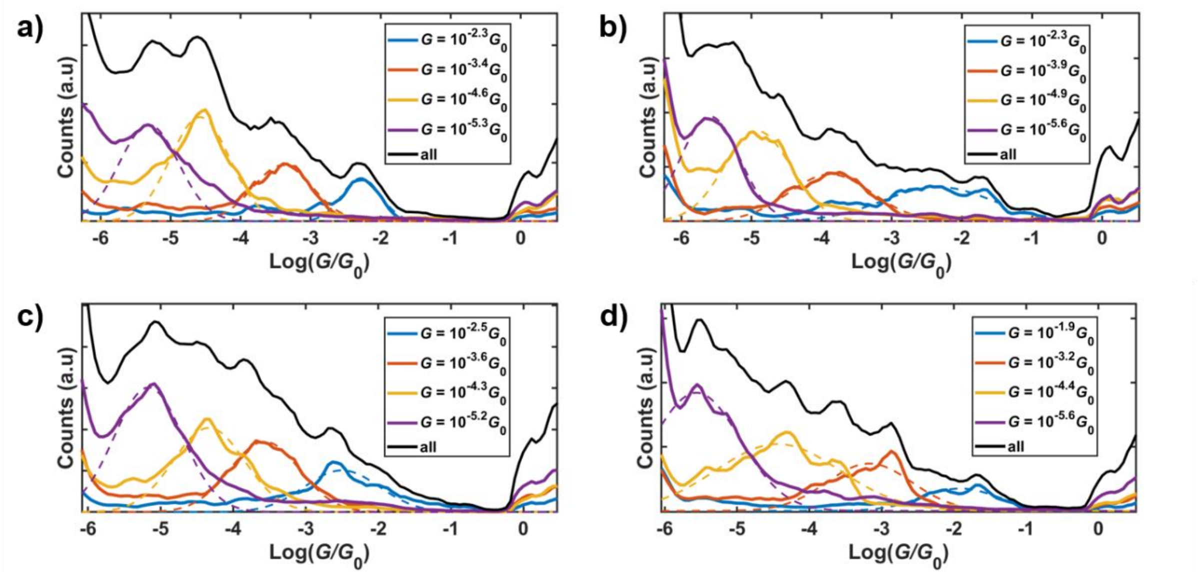

Figure S21. 1D conductance histograms of compounds (a) $\mathbf{L^p}$, (b) $\mathbf{L^m}$, (c) $\mathbf{Pd^p}$ and (d) $\mathbf{Pd^m}$ respectively, measured in air conditions; separated in conductance classes by colors (the highest conductance peak in blue, then the second highest in red, the main conductance peak in yellow and the lowest conductance peak in purple), each of them fitted to a Gaussian distribution displayed by dashed lines. The black histogram is the total considering all the classes. The legend shows the mean *G* value for each class.

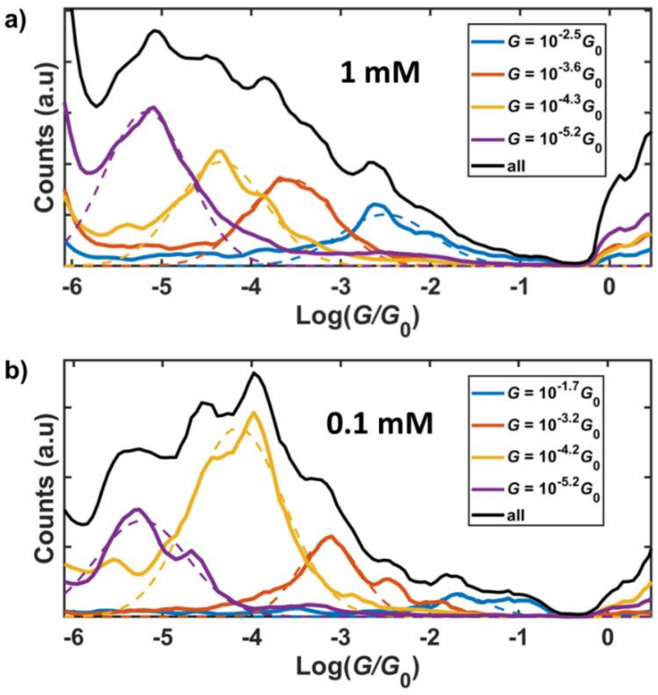

Figure S22. Comparison of 1D conductance histograms of compound **Pd^{p}** measured in air from an (a) ~ 1 mM and (b) ~ 0.1 mM DCM solution; separated in conductance classes by colors (the highest conductance peak in blue, then the second highest in red, the main conductance peak in yellow and the lowest conductance peak in purple), each of them fitted to a Gaussian distribution displayed by dashed lines. The black histogram is the total considering all the classes. The legend shows the mean *G* value for each class.

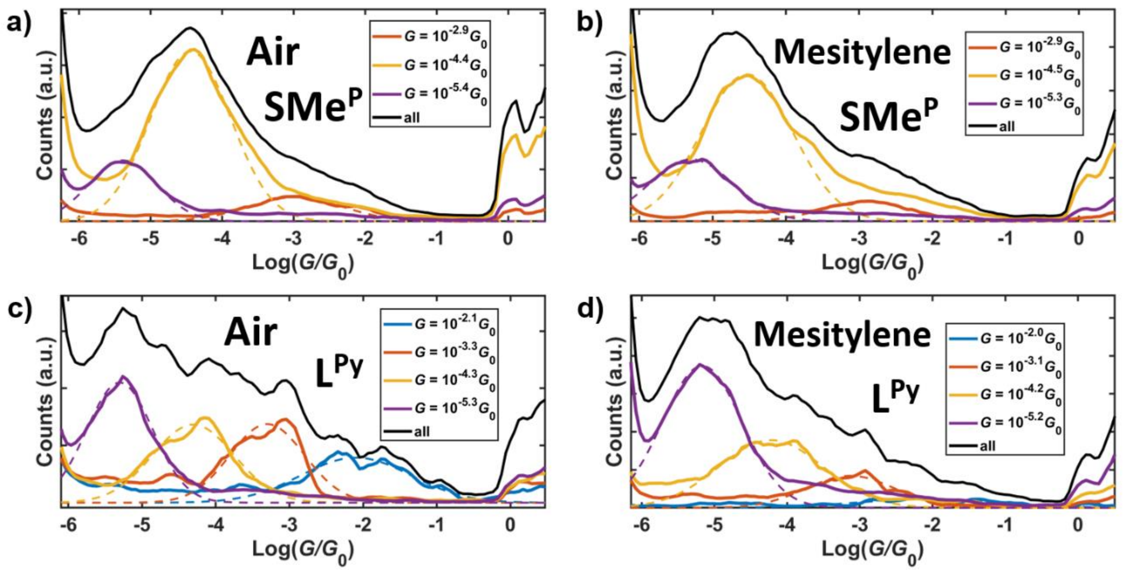

Figure S23. Comparison of 1D conductance histograms of compounds (a,b) **SMe**$^{\mathbf{p}}$ and (c,d) **L**$^{\mathbf{py}}$, measured in air and solution respectively; separated in conductance classes by colors (the highest conductance peak in blue, then the second highest in red, the main conductance peak in yellow and the lowest conductance peak in purple), each of them fitted to a Gaussian distribution displayed by dashed lines. The black histogram is the total considering all the classes. The legend shows the mean *G* value for each class.

2. 2D Conductance-distance histograms

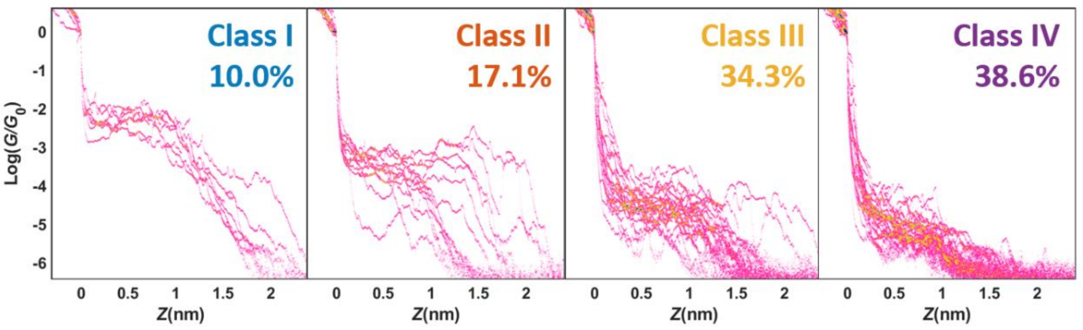

Figure S24. 2D conductance-distance histograms of compound **L**$^{\mathbf{p}}$ for all the measurements with a molecular response recorded in air conditions, separated by conductance classes. Inset: relative percentage of the total selected traces contributing to each conductance class.

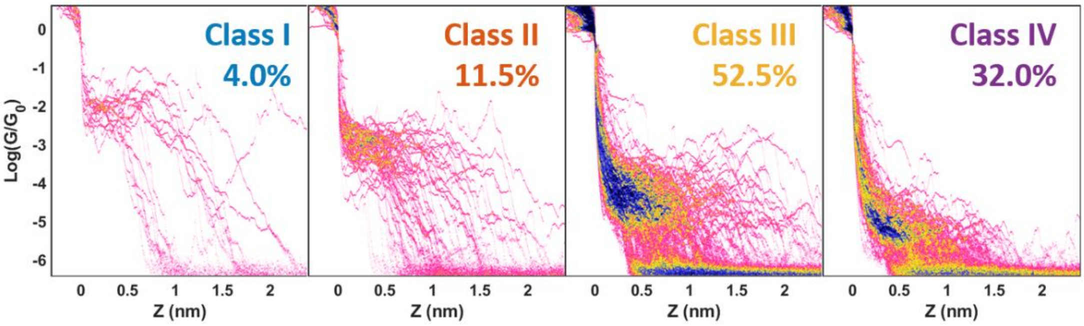

Figure S25. 2D conductance-distance histograms of compound **L**$^{\mathbf{p}}$ for all the measurements with a molecular response recorded in solution, separated by conductance classes. Inset: relative percentage of the total selected traces contributing to each conductance class.

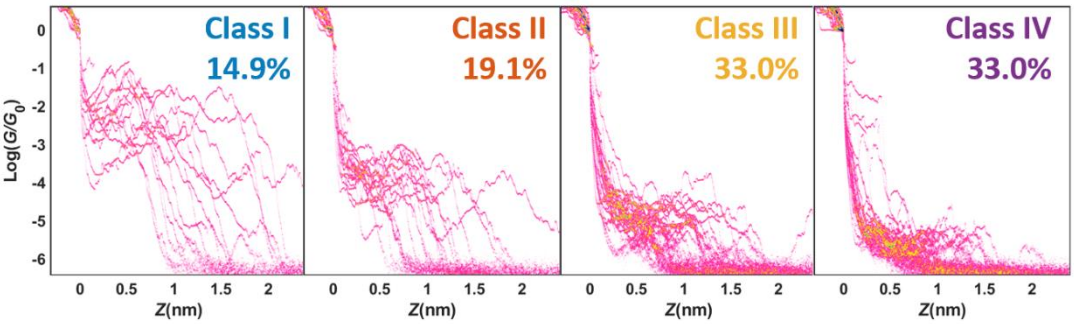

Figure S26. 2D conductance-distance histograms of compound **L**$^{\mathbf{m}}$ for all the measurements with a molecular response recorded in air conditions, separated by conductance classes. Inset: relative percentage of the total selected traces contributing to each conductance class.

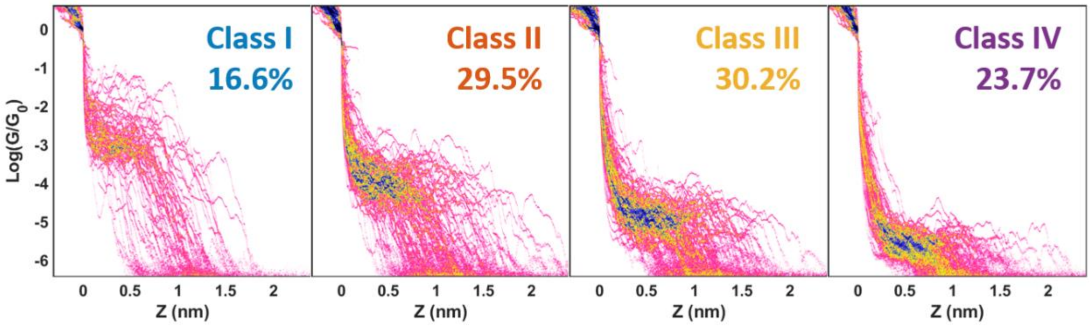

Figure S27. 2D conductance-distance histograms of compound **L^{m}** for all the measurements with a molecular response recorded in solution, separated by conductance classes. Inset: relative percentage of the total selected traces contributing to each conductance class.

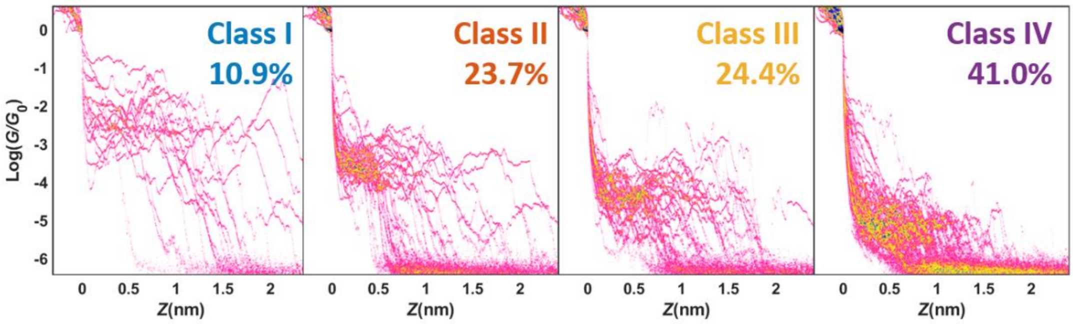

Figure S28. 2D conductance-distance histograms of compound **Pd^{p}** for all the measurements with a molecular response recorded in air conditions, separated by conductance classes. Inset: relative percentage of the total selected traces contributing to each conductance class.

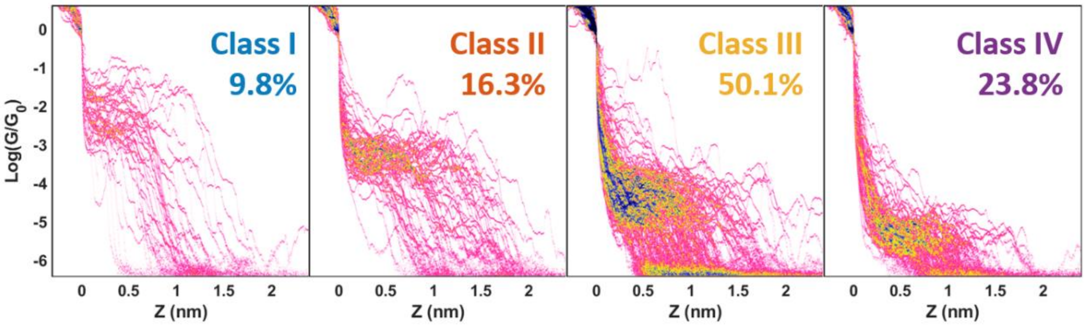

Figure S29. 2D conductance-distance histograms of compound **Pd^{p}** for all the measurements with a molecular response recorded in solution, separated by conductance classes. Inset: relative percentage of the total selected traces contributing to each conductance class.

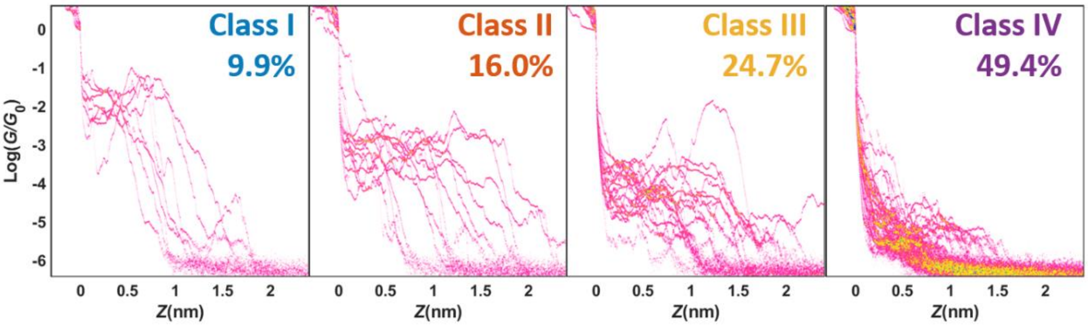

Figure S30. 2D conductance-distance histograms of compound **Pd^{m}** for all the measurements with a molecular response recorded in air conditions, separated by conductance classes. Inset: relative percentage of the total selected traces contributing to each conductance class.

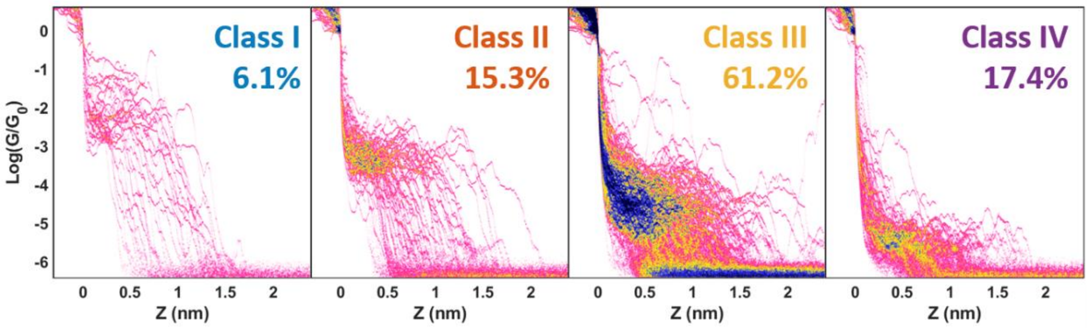

Figure S31. 2D conductance-distance histograms of compound **Pd^{m}** for all the measurements with a molecular response recorded in solution, separated by conductance classes. (inset) relative percentage of the total selected traces contributing to each conductance class.

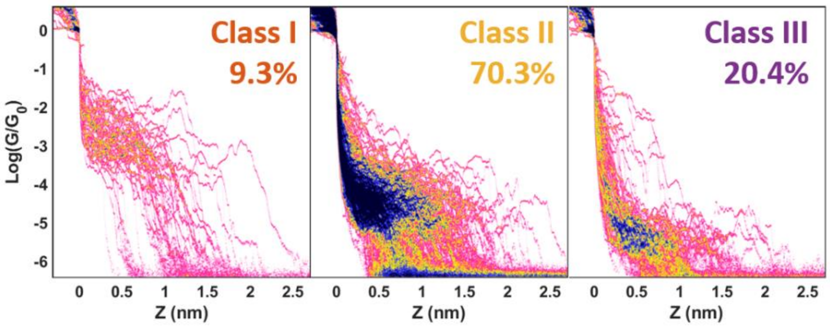

Figure S32. 2D conductance-distance histograms of compound **SMe^{p}** for all the measurements with a molecular response recorded in air conditions, separated by conductance classes. Inset: relative percentage of the total selected traces contributing to each conductance class.

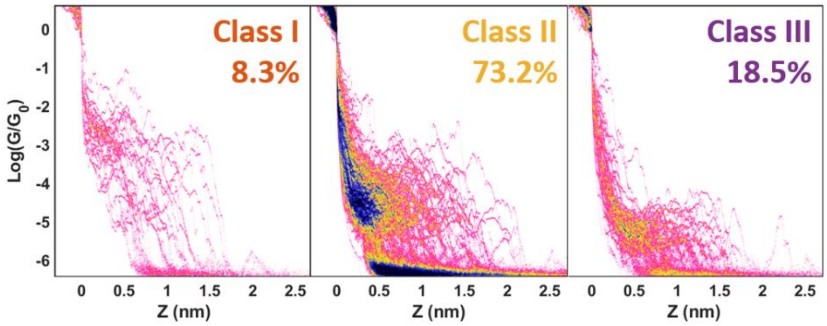

Figure S33. 2D conductance-distance histograms of compound **SMe^p** for all the measurements with a molecular response recorded in solution, separated by conductance classes. Inset: relative percentage of the total selected traces contributing to each conductance class.

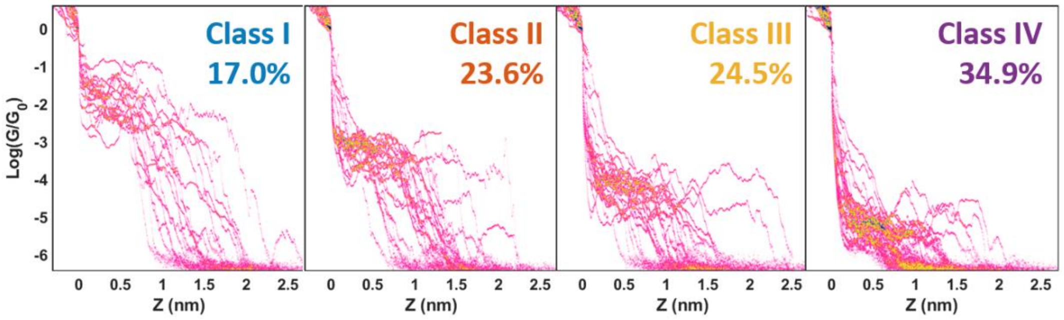

Figure S34. 2D conductance-distance histograms of compound **L^py** for all the measurements with a molecular response recorded in air conditions, separated by conductance classes. Inset: relative percentage of the total selected traces contributing to each conductance class.

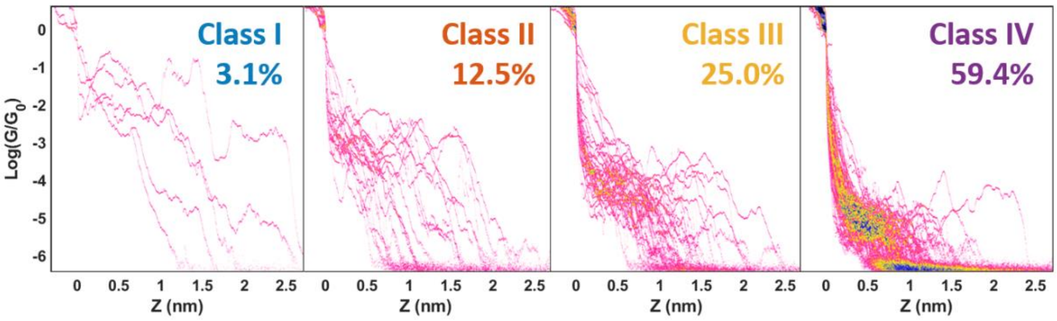

Figure S35. 2D conductance-distance histograms of compound $\mathbf{L^{py}}$ for all the measurements with a molecular response recorded in solution, separated by conductance classes. Inset: relative percentage of the total selected traces contributing to each conductance class.

3. Junction length determination

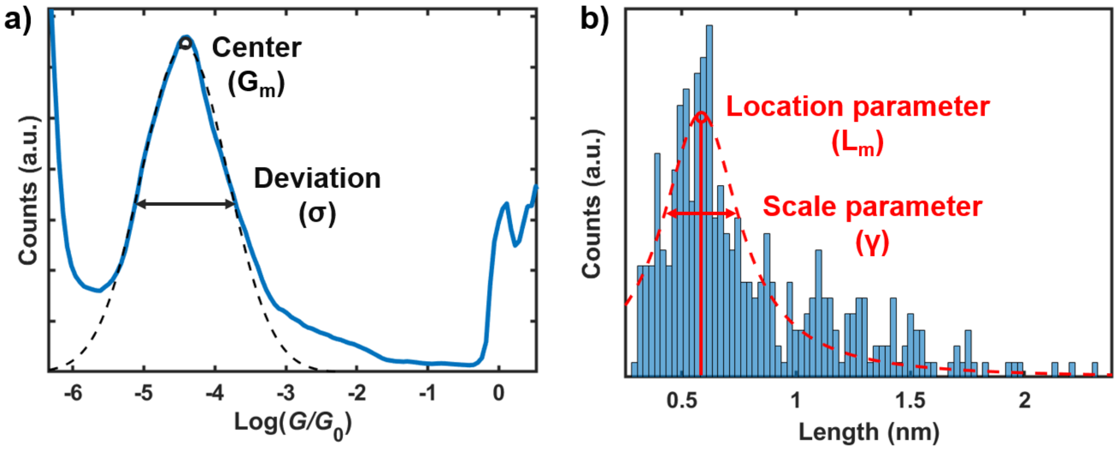

Figure S36. (a) Gaussian fitting of the 1D conductance histogram of the Class II of compound $\mathbf{SMe^{p}}$ measured in air. (b) Length histogram obtained with the counts as the points with higher distance value within $G_m \pm 2\sigma$ for each trace. Lorentzian fitting as a dashed line, where the break-off distance is the mean value of all the points above the location parameter plus the scale parameter.

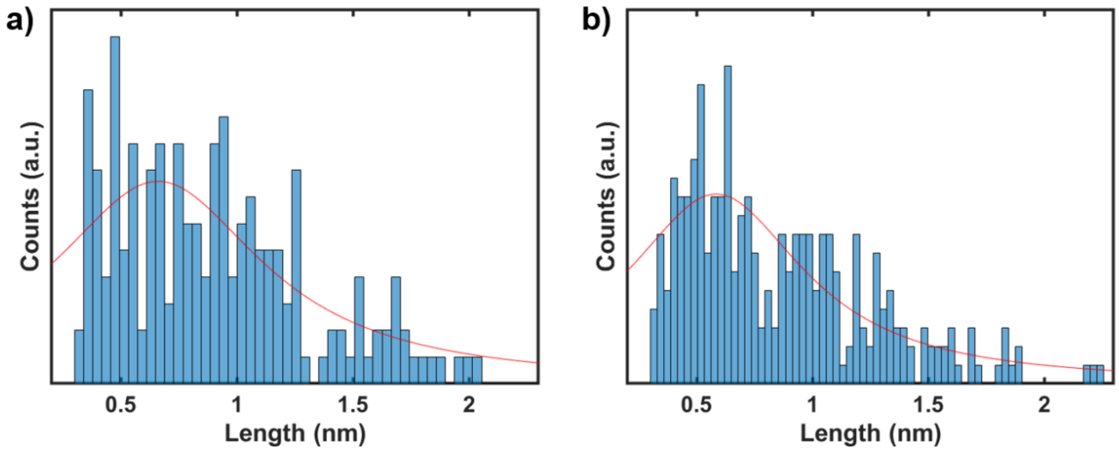

Figure S37. Length histogram obtained with the counts as the points with higher distance value within G_m±2σ for each trace of the Class III of compounds (a) **Pd^p** and (b) **Pd^m** measured in solution. Lorentzian fitting as a solid line.

Table S3. The mean length of the plateaus for all **L^p**, **L^m**, **Pd^p** and **Pd^m** recorded in air

**Molecule**	**Mean length of each class (nm)**			
	**C I**	**C II**	**C III**	**C IV**
**L^p**	1.09 ± 0.29	1.15 ± 0.27	1.34 ± 0.27	1.00 ± 0.21
**L^m**	1.21 ± 0.36	0.62 ± 0.30	0.78 ± 0.44	1.30 ± 0.16
**Pd^p**	1.11 ± 0.27	1.12 ± 0.41	1.45 ± 0.38	1.45 ± 0.36
**Pd^m**	0.53 ± 0.22	1.07 ± 0.27	0.96 ± 0.32	1.04 ± 0.27

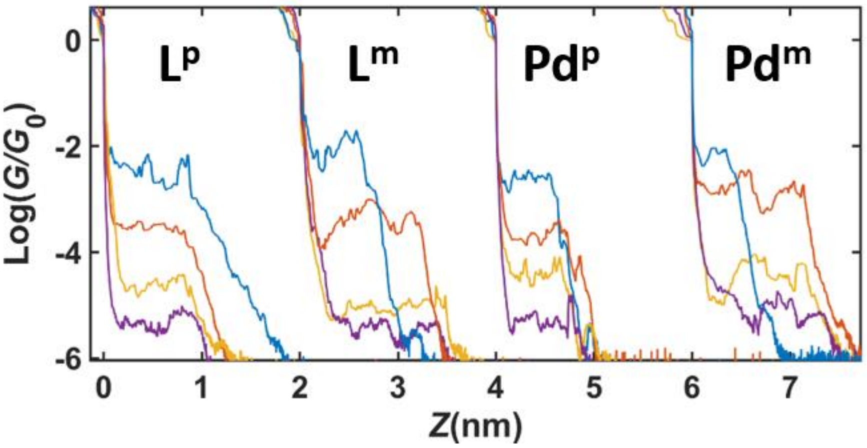

Figure S38. Examples of individual traces representative of each conductance class of the four compounds measured in air.

4. Conductance switching

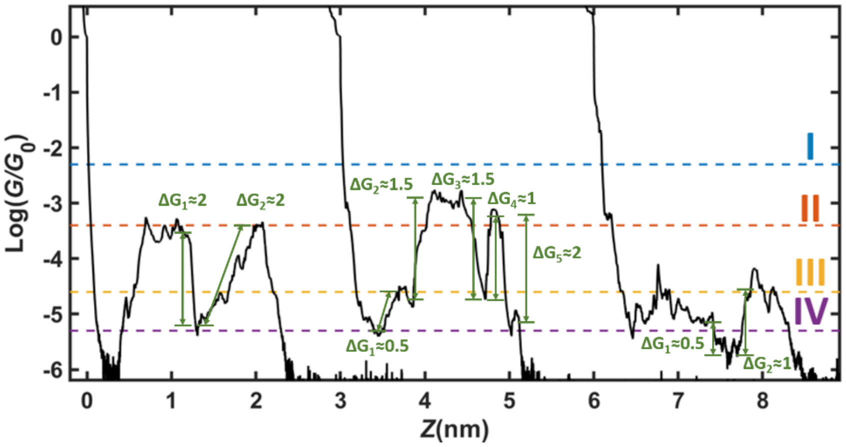

Figure S39. Examples of individual traces extracted from measurements of $\mathbf{L^p}$ in air conditions exhibiting the conductance switching behavior between the different conductance values represented as dashed lines with the color of each conductance class. The conductance changes are marked with lines indicating the approximated orders of magnitude as ΔG.

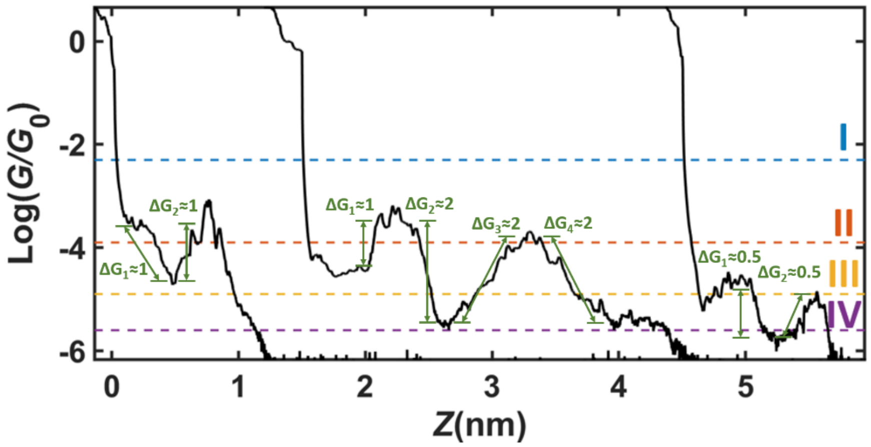

Figure S40. Examples of individual traces extracted from measurements of $\mathbf{L^m}$ in air conditions exhibiting the conductance switching behavior between the different conductance values represented as dashed lines with the color of each conductance class. The conductance changes are marked with lines indicating the approximated orders of magnitude as ΔG.

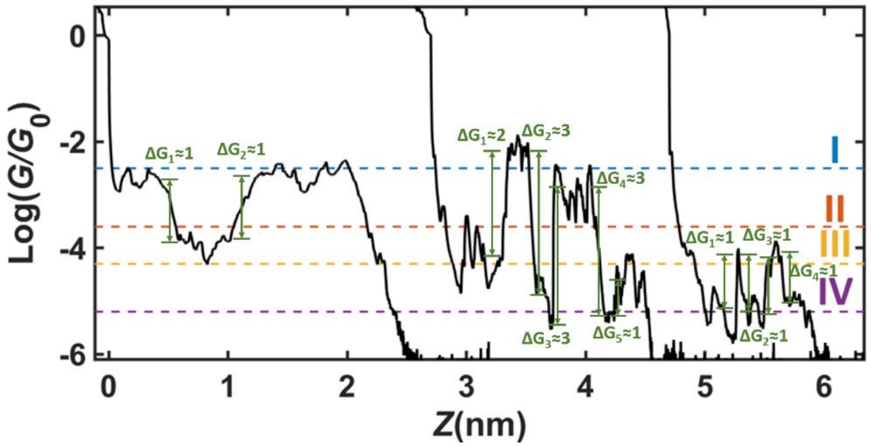

Figure S41. Examples of individual traces extracted from measurements of $\mathbf{Pd^p}$ in air conditions exhibiting the conductance switching behavior between the different conductance values represented as dashed lines with the color of each conductance class. The conductance changes are marked with lines indicating the approximated orders of magnitude as ΔG.

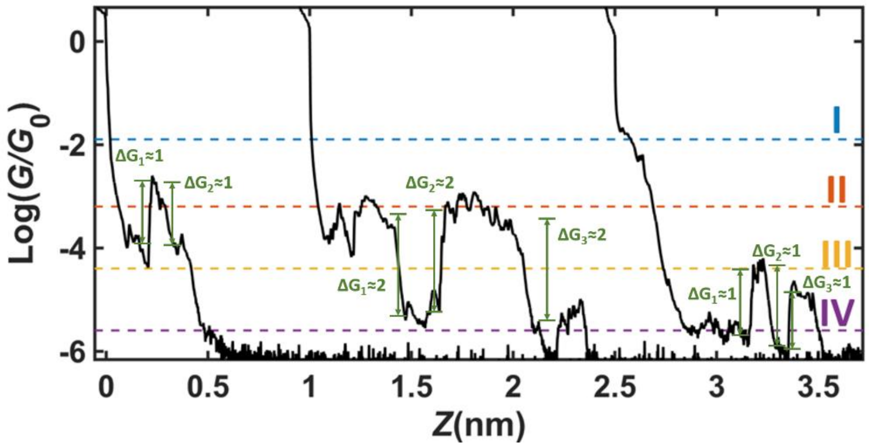

Figure S42. Examples of individual traces extracted from measurements of **Pd^m^** in air conditions exhibiting the conductance switching behavior between the different conductance values represented as dashed lines with the color of each conductance class. The conductance changes are marked with lines indicating the approximated orders of magnitude as ΔG.

d) XPS results

Binding energies were calibrated according to the C1s peak at 284.6 eV. The C 1s peak was chosen, rather than for example Au 4f, for consistency with all the samples (powders and SAMs) although possible inaccuracies of using the C 1s binding energy have been reviewed.[7] XPS peak fitting was carried out using the CASA software with the Shirley BG type.[8]

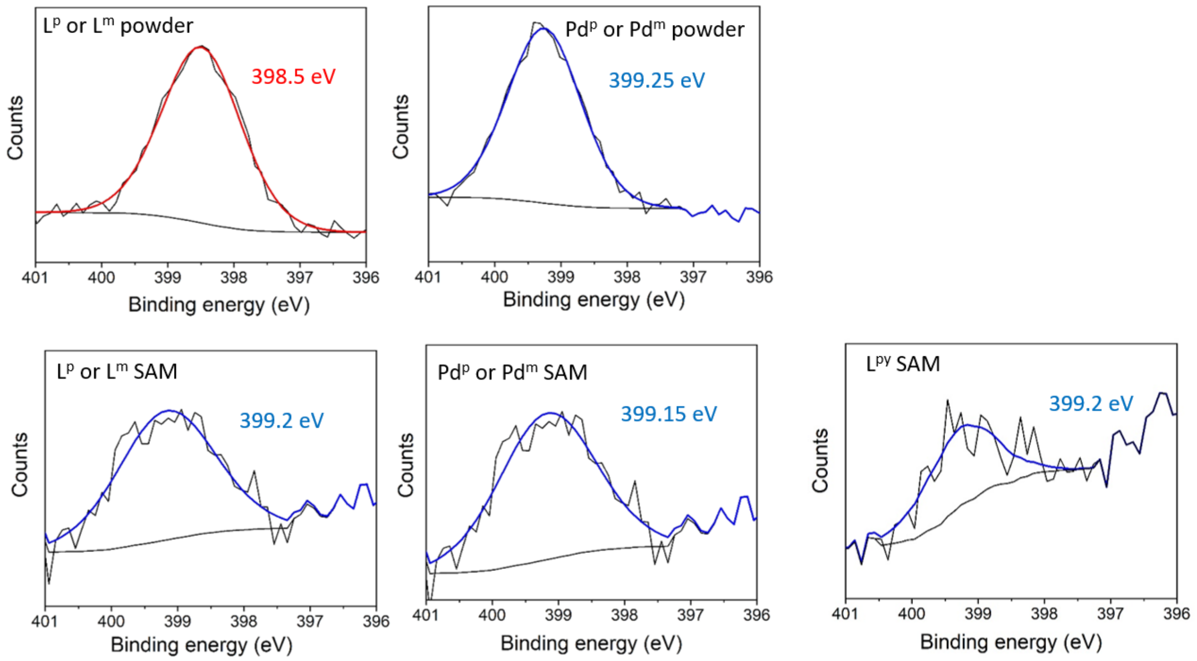

Figure S43. XPS spectra in the N1s region corresponding to **L**$^{\mathbf{p}}$, **L**$^{\mathbf{m}}$, **Pd**$^{\mathbf{p}}$ and **Pd**$^{\mathbf{m}}$ powders and SAMs of **L**$^{\mathbf{p}}$, **L**$^{\mathbf{m}}$, **Pd**$^{\mathbf{p}}$, **Pd**$^{\mathbf{m}}$ and **L**$^{\mathbf{py}}$. The same XPS spectra were obtained for **L**$^{\mathbf{p}}$ and **L**$^{\mathbf{m}}$ powders; **Pd**$^{\mathbf{p}}$ and **Pd**$^{\mathbf{m}}$ powders, and for their respective SAMs. Therefore, only one spectrum is shown in each panel.

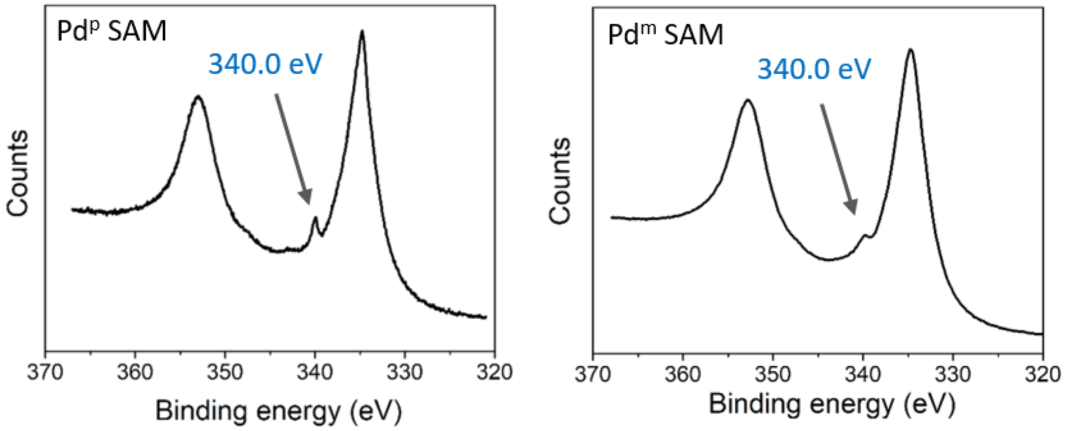

Figure S44. XPS spectra in the Au4d/Pd3p region corresponding to **Pd^{p}** and **Pd^{m}** SAMs where clearly a peak attributed to Pd(0), Pd3p$_{3/2}$, is observed at 340.0 eV. Note that the Pd3p$_{1/2}$ peak is obscured by the Au4d$_{5/2}$ peak due to the gold substrate.

e) Theoretical Calculations

The optimum geometry of each of the molecules was calculated using the density functional code SIESTA.[9] These used a double-zeta polarized (DZP) basis set defined by a confining cut-off of 0.008 Rydbergs, norm conserving pseudopotentials, an energy cut-off of 150 Rydbergs and the generalized gradient approximation (GGA) method to describe the exchange correlation functional. All forces on the atoms were relaxed to a force tolerance of 0.01 eV/Å. The molecule was then contacted to gold electrodes to form the molecular junction. The gold electrodes were modelled as 6 layers of (111) gold each containing 25 atoms which are terminated by a pyramid of 13 gold atoms to mimic an STM tip. A double-zeta basis was used to describe the gold atoms, and a Hamiltonian describing this extended molecule was extracted using SIESTA. The zero bias transmission coefficient $T(E)$, conductance G and the Seebeck coefficient S were calculated using the quantum transport code Gollum.[10] During the optimization apical Au atoms were also relaxed. In each simulation, a pyramid of gold atoms was used as a tip, to model the last few atoms resulting from the breaking of the junction, as discussed, for example, in ref.[11].

In the case of the ligands **L^{p}** and **L^{m}** the chemical structure makes it possible for the geometry to change as the nitrogen atom of the pyridine unit can either point inwards to the center of the molecule or point outwards. This is important as pyridine can act as an anchor unit with gold attaching to the nitrogen atom. Three possible conformations of **L^{p}** are identified due to the orientation of the pyridine: 1 – both pyridines pointing inwards, 2 – both pyridines pointing outwards and 3 – one pyridine pointing in and one pointing out. The second important orientation is that of the methyl group, which has its minimum energy when it is

aligned with the plane of the pyridine ring. Therefore, for molecule **L^p^** the methyl group can point left or right, and this leads to 3 possible orientations; A – left and right, B – right and right and C right and left. We also consider the case where the methyl groups are rotated out of the plane by 90° which is shown as geometry D. The corresponding relaxed optimum geometry of each of the 12 isomers can be seen in Figure S45, along with the ground state energy Eg relative to the minimum energy 0 eV. In this series, the geometry 3C has the lowest energy, which corresponds to the pyridines pointing in opposite directions and may be due to an interaction between the nitrogen and the hydrogen on the opposite ring. Note that the energy difference to 3A and 3B is very small (0.01 eV), which indicates that the orientation of the methyl groups is less important.

Rotamers of L^p

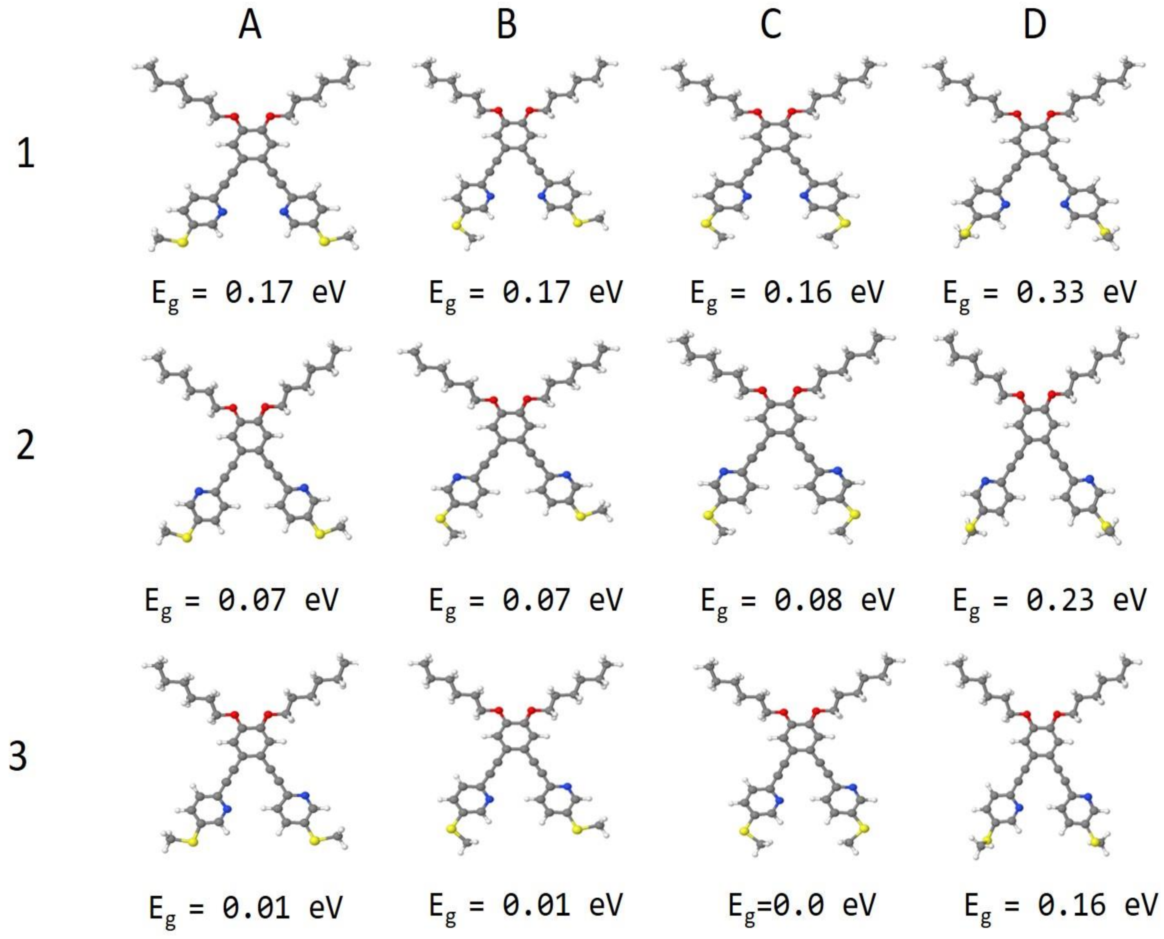

Figure S45. Possible rotamers of the $\mathbf{L^p}$ molecule including their ground state energy relative to the global minimum 0 eV.

In Figure S46, the process is repeated for the $\mathbf{L^m}$ molecule. Again, there are the same three conformations of the pyridine rings (1-3), however due to symmetry, there is an extra orientation D for the methyl group in the case of geometry 3. Orientation E again corresponds to the methyl groups rotated 90° out of the plane. The lowest energy isomer is 3C with the pyridine rings pointing in opposite directions.

Rotamers of $\mathbf{L^m}$

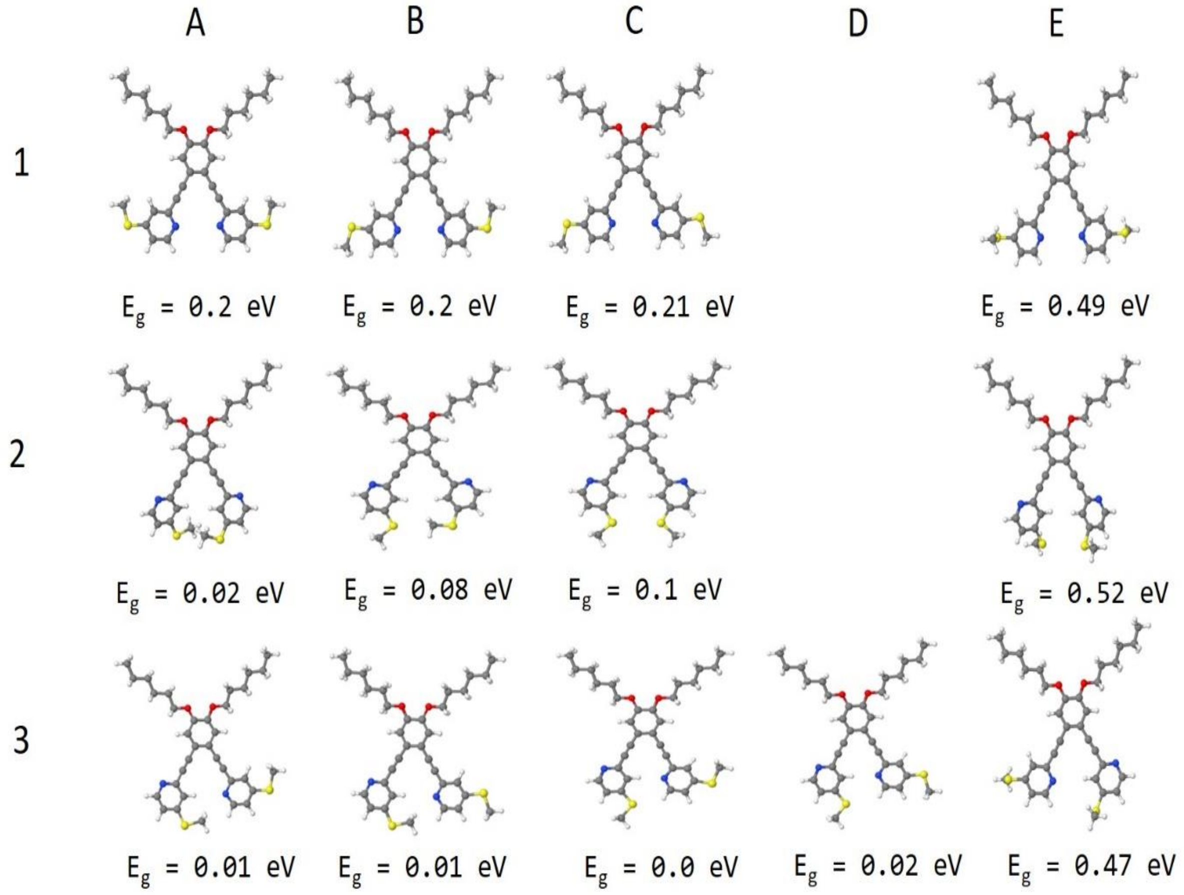

Figure S46. Possible rotamers of the $\mathbf{L^m}$ molecule including their ground state energy relative to the global minimum 0 eV.

In the case of the complex molecules **Pd^p** and **Pd^m** (Figure S47) the orientation of the pyridine rings is fixed, so only the methyl groups are free to move. As before, four different positions A-D are assumed, where for **Pd^p** A – left and right, B – left and left, C – right and left and D – methyl rotated 90° out of the plane. For **Pd^m** the orientations are A – up and up, B – up and down, C – down and down and D – rotated 90° to the plane. The energy difference from the minimum is small except in the case of the isomer D.

Geometries of Pd^p and Pd^m

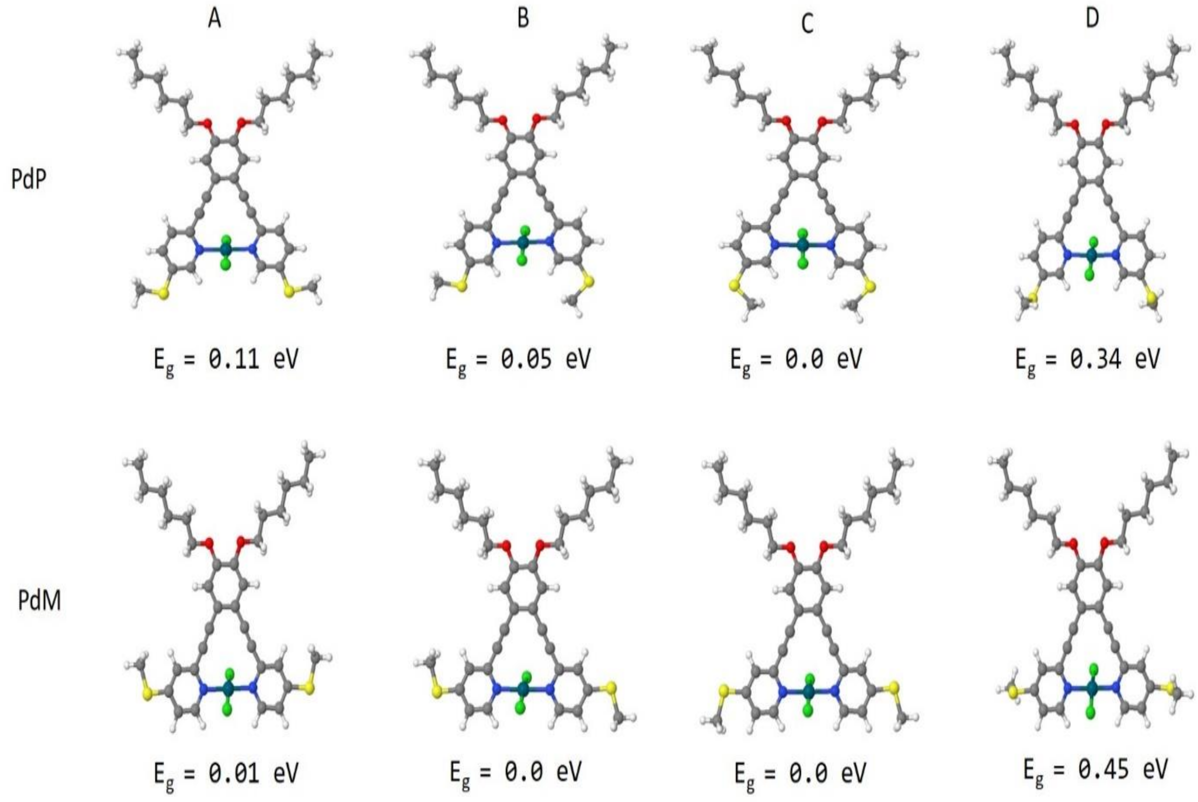

Figure S47. Possible isomers of the **Pd^p** and **Pd^m** molecules including their ground state energy relative to the global minimum 0 eV.

Electronic Structure of molecules

As described in ref.[9], the ionization potential (IP) was calculated using the formula IP=E(N-1)-E(N) and the electron affinity using the formula EA=E(N)-E(N+1) where E is the ground state energy of the molecule and N is the number of electrons

Table S4. HOMO and LUMO energy levels and Ionization Potential and Electron Affinity of the $\mathbf{L^p}$ molecules shown in Figure S45

	HOMO (eV)	LUMO (eV)	IP (eV)	EA (eV)
L^p_1A	-4.66	-2.54	6.17	0.98
L^p_1B	-4.67	-2.55	6.19	0.99
L^p_1C	-4.69	-2.58	6.21	1.01
L^p_1D	-5.03	-2.88	6.61	1.32
L^p_2A	-4.77	-2.67	6.28	1.11
L^p_2B	-4.77	-2.67	6.28	1.11
L^p_2C	-4.77	-2.67	6.29	1.10
L^p_2D	-5.11	-3.00	6.68	1.43
L^p_3A	-4.70	-2.61	6.22	1.03
L^p_3B	-4.72	-2.64	6.25	1.05
L^p_3C	-4.71	-2.65	6.26	1.05
L^p_3D	-5.05	-2.96	6.63	1.36

Table S5. HOMO and LUMO energy levels and Ionization Potential and Electron Affinity of the $\mathbf{L^m}$ molecules shown in Figure S46

	HOMO (eV)	LUMO (eV)	IP (eV)	EA (eV)
L^m_1A	-4.92	-2.67	6.56	1.07
L^m_1B	-4.90	-2.64	6.55	1.04
L^m_1C	-4.87	-2.60	6.54	1.01
L^m_1E	-5.01	-2.82	6.63	1.24
L^m_2A	-5.04	-2.78	6.75	1.16
L^m_2B	-4.99	-2.76	6.67	1.16
L^m_2C	-4.93	-2.69	6.63	1.09
L^m_2E	-5.054	-2.84	6.68	1.24
L^m_3A	-4.99	-2.78	6.64	1.16
L^m_3B	-4.95	-2.74	6.62	1.13
L^m_3C	-4.90	-2.68	6.59	1.05
L^m_3D	-4.89	-2.65	6.58	1.04
L^m_3E	-5.05	-2.89	6.67	1.29

Table S6. HOMO and LUMO energy levels and Ionization Potential and Electron Affinity of the **Pd**^{p} molecules shown in Figure S47

	HOMO (eV)	LUMO (eV)	IP (eV)	EA (eV)
**Pd**^{p}_A	-4.43	-2.96	6.29	1.41
**Pd**^{p} B	-4.49	-2.99	6.34	1.44
**Pd**^{p} C	-4.55	-3.02	6.37	1.47
**Pd**^{p} D	-4.61	-3.34	6.61	1.78

Table S7. HOMO and LUMO energy levels and Ionization Potential and Electron Affinity of the **Pd**^{m} molecules shown in Figure S47

	HOMO (eV)	LUMO (eV)	IP (eV)	EA (eV)
**Pd**^{m}_A	-4.40	-3.08	6.47	1.51
**Pd**^{m} B	-4.40	-3.05	6.44	1.47
**Pd**^{m} C	-4.40	-3.01	6.43	1.44
**Pd**^{m} D	-4.61	-3.28	6.62	1.71

HOMO and LUMO orbitals

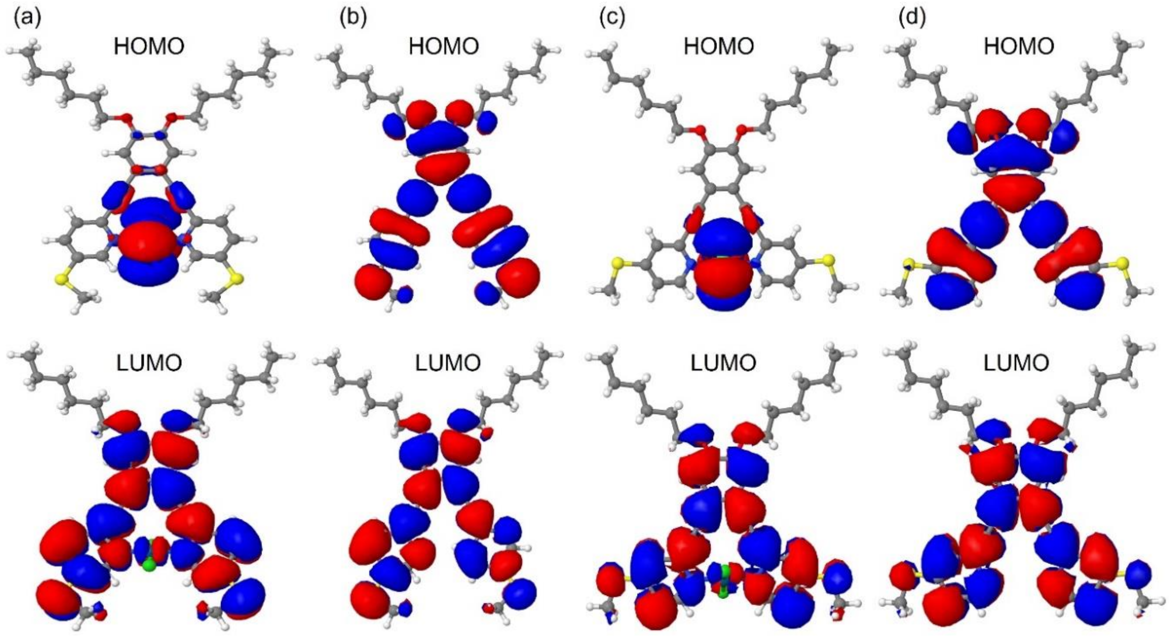

Figure S48. HOMO and LUMO orbitals of (a) **Pd^{p}**, (b) **L^{p}**, (c) **Pd^{m}** and (d) **L^{m}**

Pendulum motion about Pd atom

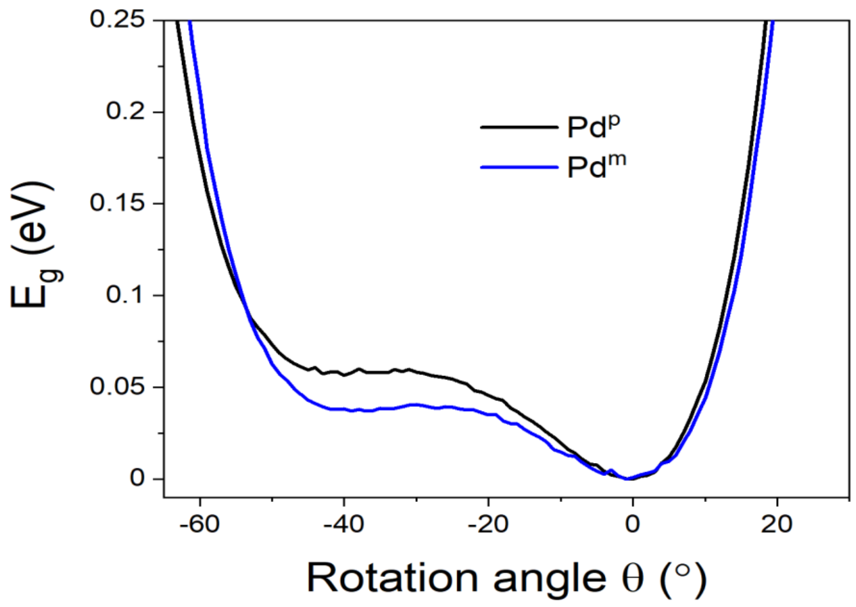

Figure S49. Ground state energy as a function of rotation angle θ about the equilibrium position 0° for **Pd^p** and **Pd^m**.

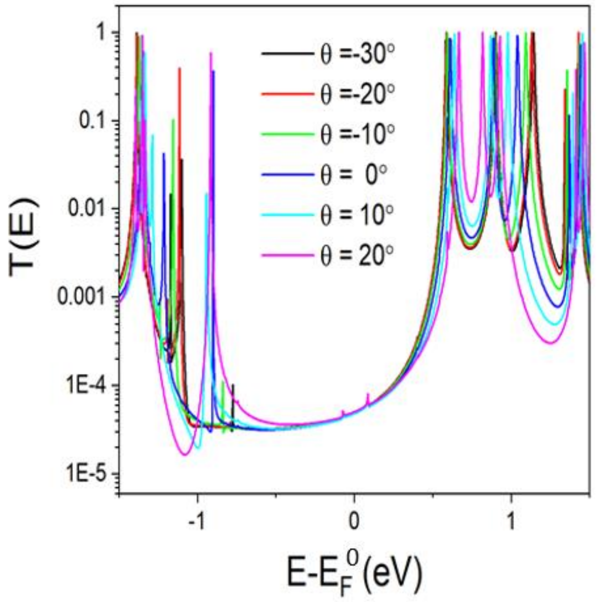

Figure S50. T(E) against energy E for rotation angle θ about the equilibrium position 0° in molecule **Pd^p**.

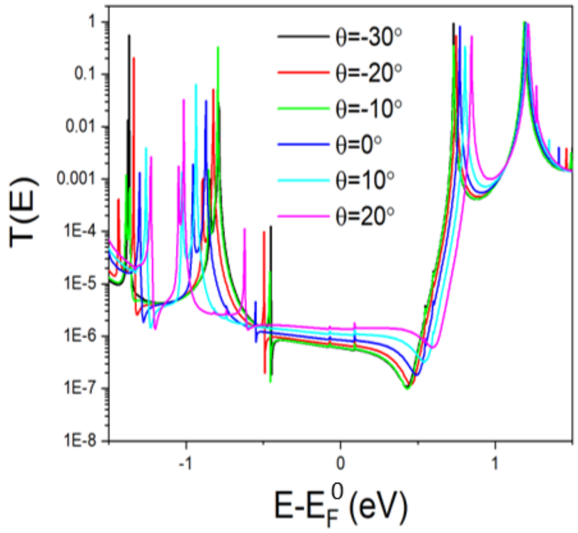

Figure S51. T(E) against energy E for rotation angle θ about the equilibrium position 0° in molecule **Pd^m**.

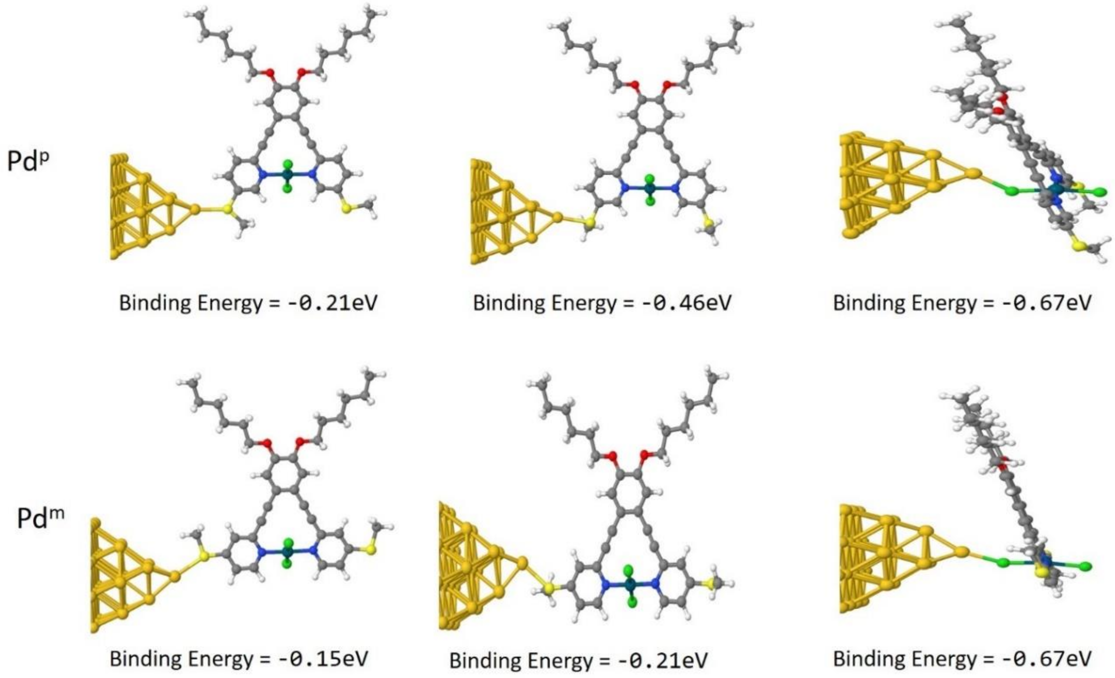

Figure S52. Binding energies of **Pd^p** and **Pd^m** molecule to a gold electrode.

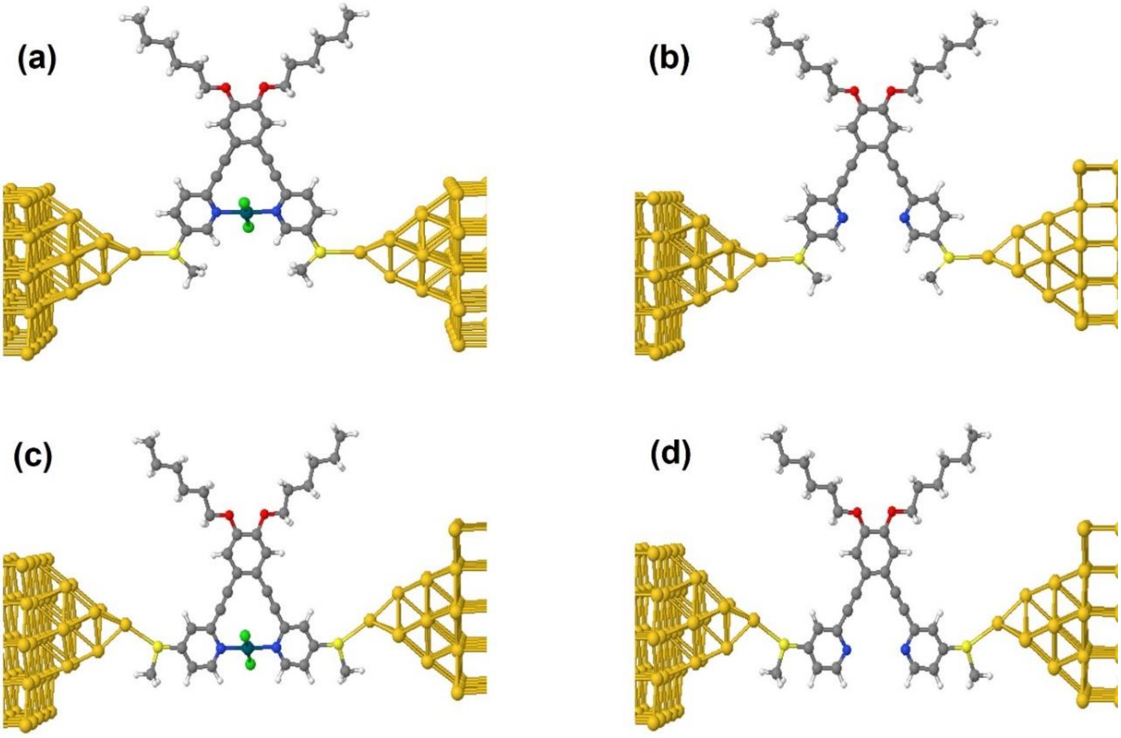

Figure S53. Molecular junction geometry for (a) **Pd^p**, (b) **L^p**, (c) **Pd^m** and (d) **L^m**.

References

1. Li, J.; Lynch, M. P.; DeMello, K. L.; Sakya, S. M.; Cheng, H.; Rafka, R. J.; Bronk, B. S.; Jaynes, B. H.; Kilroy, C.; Mann, D. W.; Haven, M. L.; Kolosko, N. L.; Petras, C.; Seibel, S. B.; Lund, L. A. In vitro and in vivo profile of 2-(3-di-fluoromethyl-5-phenylpyrazol-1-yl)-5-methanesulfonylpyridine, a potent, selective, and orally active canine COX-2 inhibitor. *Bioorganic & Medicinal Chemistry* **2005,** 13 (5), 1805-1809 DOI: 10.1016/j.bmc.2004.11.048.

2. Dai, C.; Yuan, Z.; Collings, J. C.; Fasina, T. M.; Thomas, R. L.; Roscoe, K. P.; Stimson, L. M.; Yufit, D. S.; Batsanov, A. S.; Howard, J. A. K.; Marder, T. B. Crystal engineering with p-substituted 4-ethynylbenzenes using the C–H···O supramolecular synthon. *CrystEngComm* **2004,** 6 (32), 184-188 DOI: 10.1039/B404502E.

3. Prabhath, M. R. R.; Romanova, J.; Curry, R. J.; Silva, S. R. P.; Jarowski, P. D. The Role of Substituent Effects in Tuning Metallophilic Interactions and Emission Energy of Bis-4-(2-pyridyl)-1,2,3-triazolatoplatinum(II) Complexes. *Angewandte Chemie International Edition* **2015,** 54 (27), 7949-7953 DOI: 10.1002/anie.201502390.

4. Fukushima, S.; Ashizawa, M.; Kawauchi, S.; Michinobu, T. Strain-Promoted Double Azide Addition to Octadehydrodibenzo[12]annulene Derivatives. *Helvetica Chimica Acta* **2019,** 102 (4), e1900016 DOI: 10.1002/hlca.201900016.

5. Sheldrick, G. M. *SHELXL: Suite of Programs for Crystal Structure Analysis,* Tammanstrasse 4: Gottingen, 1998.

6. Dolomanov, O. V.; Bourhis, L. J.; Gildea, R. J.; Howard, J. A. K.; Puschmann, H. OLEX2: a complete structure solution, refinement and analysis program. *Journal of Applied Crystallography* **2009,** 42, 339-341 DOI: 10.1107/s0021889808042726.

7. Greczynski, G.; L. Hultman, L. X-ray photoelectron spectroscopy: towards reliable binding energy referencing. *Prog. Mater. Sci*. **2020**, 107, 100591 doi.org/10.1016/j.pmatsci.2019.100591.

8. Major, G. H.; Fairley, N.; Sherwood, P. M. A.; Linford, M. R.; Terry, J.; Fernandez, V.; Artyushkova, K. Practical guide for curve fitting in x-ray photoelectron spectroscopy. *J. Vac. Sci. Technol. A* **2020**, 38, 061203 DOI: 10.1116/6.0000377.

9. Soler, J. M.; Artacho, E.; Gale, J. D.; Garcia, A.; Junquera, J.; Ordejon, P.; Sanchez-Portal, D. The SIESTA method for ab initio order-N materials simulation. *Journal of Physics: Condensed Matter* **2002,** 14 (11), 2745-2779 DOI: 10.1088/0953-8984/14/11/302.

10. Ferrer, J.; Lambert, C. J.; Garcia-Suarez, V. M.; Manrique, D. Z.; Visontai, D.; Oroszlany, L.; Rodriguez-Ferradas, R.; Grace, I.; Bailey, S. W. D.; Gillemot, K.; Sadeghi, H.; Algharagholy, L. A. GOLLUM: a next-generation simulation tool for electron, thermal and spin transport. *New Journal of Physics* **2014,** 16, 093029 DOI: 10.1088/1367-2630/16/9/093029.

11. Fu, T.; Frommer, K.; Nuckolls, C.; Venkataraman, L. Single-Molecule Junction Formation in Break-Junction Measurements. *J. Phys. Chem. Lett.* **2021**, 12 (44), 10802–10807 DOI.org/10.1021/acs.jpclett.1c03160.
